# Building and Developing a Tool (PANDEM-2 Dashboard) to Strengthen Pandemic Management: Participatory Design Study

**DOI:** 10.2196/52119

**Published:** 2025-03-05

**Authors:** Carlos Tighe, Lem Ngongalah, Alexis Sentís, Francisco Orchard, Gheorghe-Aurel Pacurar, Conor Hayes, Jessica S Hayes, Adrian Toader, Máire A Connolly

**Affiliations:** 1 Insight, SFI Research Centre for Data Analytics, University of Galway Galway Ireland; 2 Trilateral Research Ltd London United Kingdom; 3 Epidemiology Department, Epiconcept Paris France; 4 IT Department Epiconcept Paris France; 5 PMO, Modus Create Cluj Napoca Romania; 6 School of Computer Science, University of Galway Galway Ireland; 7 School of Health Sciences, University of Galway Galway Ireland; 8 Enterprise Engineering, Modus Create Cluj Napoca Romania

**Keywords:** pandemic preparedness and response, COVID-19, cross-border collaboration, surveillance, data collection, data standardization, data sharing, dashboard, IT system, IT tools

## Abstract

**Background:**

The COVID-19 pandemic exposed challenges in pandemic management, particularly in real-time data sharing and effective decision-making. Data protection concerns and the lack of data interoperability and standardization hindered the collection, analysis, and interpretation of critical information. Effective data visualization and customization are essential to facilitate decision-making.

**Objective:**

This study describes the development of the PANDEM-2 dashboard, a system providing a standardized and interactive platform for decision-making in pandemic management. It outlines the participatory approaches used to involve expert end users in its development and addresses key considerations of privacy, data protection, and ethical and social issues.

**Methods:**

Development was informed by a review of 25 publicly available COVID-19 dashboards, leading to the creation of a visualization catalog. User requirements were gathered through workshops and consultations with 20 experts from various health care and public health professions in 13 European Union countries. These were further refined by mapping variables and indicators required to fulfill the identified needs. Through a participatory design process, end users interacted with a preprototype platform, explored potential interface designs, and provided feedback to refine the system’s components. Potential privacy, data protection, and ethical and social risks associated with the technology, along with mitigation strategies, were identified through an iterative impact assessment.

**Results:**

Key variables incorporated into the PANDEM-2 dashboard included case rates, number of deaths, mortality rates, hospital resources, hospital admissions, testing, contact tracing, and vaccination uptake. Cases, deaths, and vaccination uptake were prioritized as the most relevant and readily available variables. However, data gaps, particularly in contact tracing and mortality rates, highlighted the need for better data collection and reporting mechanisms. User feedback emphasized the importance of diverse data visualization formats combining different data types, as well as analyzing data across various time frames. Users also expressed interest in generating custom visualizations and reports, especially on the impact of government interventions. Participants noted challenges in data reporting, such as inconsistencies in reporting levels, time intervals, the need for standardization between member states, and General Data Protection Regulation concerns for data sharing. Identified risks included ethical concerns (accessibility, user autonomy, responsible use, transparency, and accountability), privacy and data protection (security and access controls and data reidentification), and social issues (unintentional bias, data quality and accuracy, dependency on technology, and collaborative development). Mitigation measures focused on designing user-friendly interfaces, implementing robust security protocols, and promoting cross-member state collaboration.

**Conclusions:**

The PANDEM-2 dashboard provides an adaptable, user-friendly platform for pandemic preparedness and response. Our findings highlight the critical role of data interoperability, cross-border collaboration, and custom IT tools in strengthening future health crisis management. They also offer valuable insights into the challenges and opportunities in developing IT solutions to support pandemic preparedness.

## Introduction

### Background

The COVID-19 pandemic posed considerable challenges to European countries with widespread variation in response capacity. The COVID-19 pandemic revealed shortcomings in coordination, information sharing, and response strategies among countries, leading to fragmented approaches and delayed actions [1-3]. The lack of standardized data collection and analysis methods hindered the ability to assess the situation comprehensively, to allow data sharing and strengthen or facilitate cross-border collaboration, and to make informed decisions [4,5]. In addition, limited resources and infrastructure strained health care systems, exacerbating the impact of the COVID-19 pandemic. Lessons learned from the COVID-19 experience emphasize the critical need for enhanced preparedness and response efforts [6].

In response to the above challenges, the European Union (EU) has mandated the development and implementation of robust measures for pandemic preparedness and response across member states [7]. This mandate highlights the significance of reinforcing coordination and information exchange, as well as strengthening early detection of threats, monitoring and forecasting of diseases and resources (eg, beds and vaccines), rapid risk assessments, and the capacity of evaluating medical countermeasures (eg, therapeutics) and nonpharmaceutical interventions (eg, social distancing measures such as school closures or lockdowns). The aim is that these activities will build capacity to enhance preparedness and response capabilities to future health threats, including pandemics. Recent studies have demonstrated the potential of digital health dashboards to transform public health policy by providing real-time data analytics for rapid decision-making [8].

The PANDEM-2 IT system provides an innovative solution to address these objectives by offering a standardized and interactive platform for pandemic preparedness training and response to support decision-making on pandemic management. It also offers adaptability for addressing other potential cross-border threats. The system, which is now developed to an advanced prototype level [9,10], enables pandemic managers and stakeholders to acquire the necessary skills, knowledge, and decision-making capabilities required to respond effectively to future pandemics. Furthermore, it is designed to serve as a prototype that can be further developed or adapted to cater to different training needs. The PANDEM-2 system was tested in a 2-day functional exercise to simulate a pandemic caused by a novel strain of avian influenza. The cross-border exercise was conducted between 2 public health emergency operating centers (National Institute of Public Health & Environment, Netherlands, and Robert Koch Institute, Germany) [11]. Several EU public health and first responder agencies, including the Directorate-General for Health and Food Safety’s Early Warning Response System simulation platform, were involved in supporting roles. Qualitative and quantitative data were collected to evaluate the performance of the system and its potential as a cross-border preparedness and response tool for health emergencies. Results indicate that the PANDEM-2 IT system holds potential to become a robust training hub for pandemic management in the future, serving as a valuable resource for continuous learning and preparedness.

### Objectives

This study aimed to describe the development and potential of the PANDEM-2 dashboard and to outline the methodologies used in obtaining valuable insights and feedback from end users to further refine its development. It also describes how privacy, data protection, and security risks were considered throughout the design of the system. The lessons learned from the development and implementation of the platform can pave the way for future research and development in pandemic management and response technologies and inform the development of similar IT solutions.

## Methods

### PANDEM-2 System Architecture

#### System Design and Components

**Figure 1:**
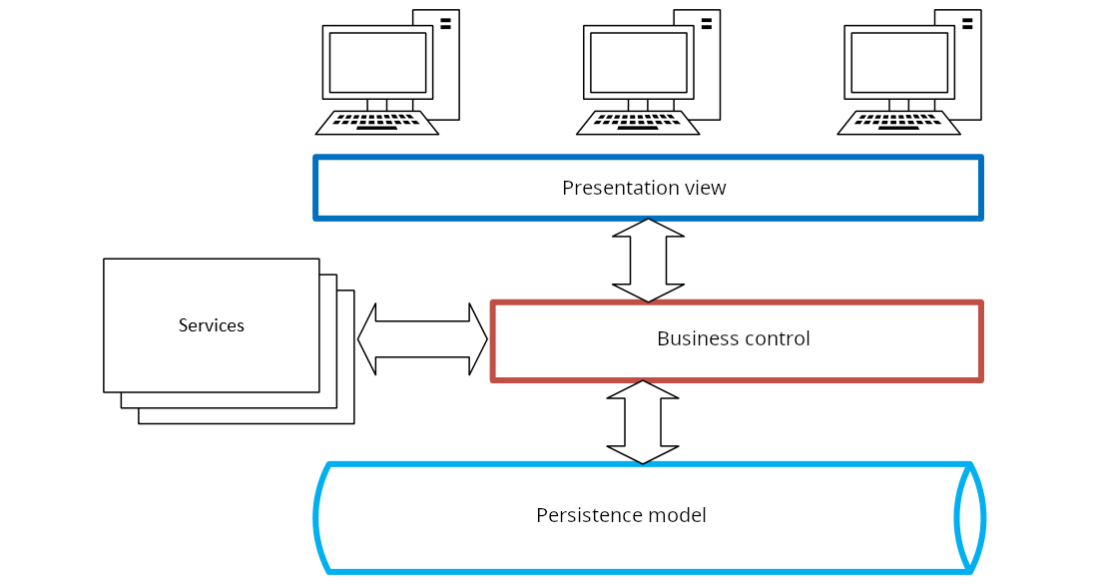
Multilayered architecture of the PANDEM-2 dashboard illustrating the interaction between the different layers, including the persistence layer (MongoDB), business layer (NodeJS), and presentation layer (Angular).

The PANDEM-2 architecture (Figure 1) is an open-source framework comprising multiple modules and components that are accessed and integrated as web services. Its design and development were guided by the selection of widely supported libraries and adherence to industry-standard development practices and principles. The platform features a multilayered architecture that facilitates communication between its components. The persistence layer, built using MongoDB [12], ensures efficient data retrieval and storage. This layer is accessed through an exposed application programming interface (API) from the NodeJS business layer [13], which facilitates interaction between the MongoDB database and other modules or external systems. NodeJS is known for its scalability and efficiency and ensures the smooth functioning of the business layer [14]. The presentation layer is developed using the Angular framework [15], which manages the user interface, providing an intuitive and interactive experience for users. Angular was chosen for its robustness, scalability, and extensive library support, which enhances the platform’s user-friendliness and maintainability. Angular is a well-maintained framework, with releases every 6 months. Its documentation is comprehensive, containing deprecation, compatibility, and breaking change policies alongside update path guides, ensuring clear upgrades and maintenance. Data visualization within the application is handled by the Highcharts JavaScript library [16]. The use of these stable and well-supported technologies underpins an architectural design that ensures the platform is scalable, adaptable to various presentation contexts, and easy to maintain.

#### Data Collection and Integration

Data within PANDEM-2 are gathered from various sources via data collectors, which can either be human-operated or automated. The PANDEM-Source module [17], an extract, transform, and load (ETL) pipeline and database was specifically developed for PANDEM-2 to facilitate the continuous collection and integration of heterogeneous surveillance data into a coherent database. PANDEM-Source uses JSON (JavaScript Object Notation)-formatted source definition files to describe each source, including format, monitoring frequency, mapping to PANDEM-2 variables, and coding systems. It performs transcoding by downloading and applying mappings between coding systems, reporting any missing codes or integration errors to a data manager who can update mappings or monitor integration progress. A key challenge was standardizing geographic locations to the Nomenclature of Territorial Units for Statistics (NUTS), a hierarchical system developed by Eurostat that divides the territory of the EU into regions for statistical and reporting purposes [18]. For natural language processing used to detect locations in social media data, we used the GeoNames database, which contains local aliases for geographic locations [19]. To reduce methodological disparities between sources, we prioritized official European Centre for Disease Prevention and Control (ECDC) data where available and relied on existing data collectors like COVID-19 Data Hub [20] when ECDC data were not available. PANDEM-Source allows data managers to tag datasets, enabling the selection of relevant data combinations for export to the PANDEM-2 dashboard. This process, while facilitated by the tool, requires human oversight. To ensure the accuracy of the data acquisition process, manual validation was performed during both the data import and dashboard implementation stages. The declarative syntax used by the tool minimized the risk of errors in data processing. A more formal validation process is currently underway and described in greater detail in a related preprint [21].

**Figure 2:**
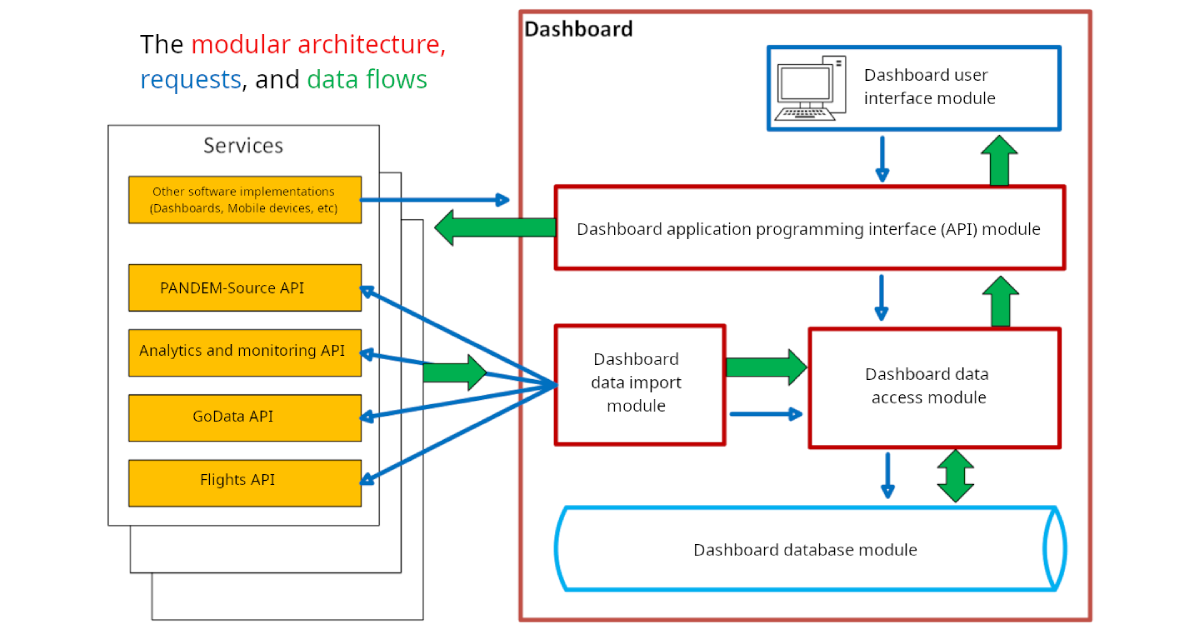
Dashboard modular architecture demonstrating the flow of data from external sources into the PANDEM-2 dashboard, showing how data is processed through various modules and application programming interfaces (APIs).

The PANDEM-Source module functions as a service with an API providing relevant and preprocessed data to the persistence layer of the dashboard. Data are then processed, exposed through a specific API, and made accessible to the front-end of the dashboard, as well as other interested data publishers (Figure 2).

The business layer in the dashboard collects data from external modules, such as the World Health Organization GoData implementation [22] and also from the Flights module [23] and the Modelling and Analytics module developed internally as part of the project. It processes these data, performing validations, adaptations, and enrichment based on formulas, to prepare them for display in the presentation layer.

#### Security Aspects of the PANDEM-2 Dashboard

The PANDEM-2 dashboard infrastructure is secured using Amazon Web Services (AWS) Cloud Security [24]. The AWS environment is monitored using AWS CloudWatch and CloudTrail, ensuring General Data Protection Regulation (GDPR) compliance [25]. Several other AWS services were used to enhance privacy and control network access, including Amazon GuardDuty for continuous threat monitoring [26] and AWS Secret Manager and Key Management Services for data protection [27]. Data are encrypted in transit with Transport Layer Security across the AWS services, and secure sockets layer and transport layer security certificates, are managed with AWS Certificate Manager. A summary of the PANDEM-2 security aspects can be found in Multimedia Appendix 1. The project only hosted publicly available aggregated or simulated data, which addressed data protection concerns for the duration of the project.

### Dashboard Design and Development

#### Exploratory Research and Design Rationale: Why a Dashboard Approach?

A dashboard is a visual display of key analytical indicators arranged on a single screen, enabling real-time monitoring and quick assessment [28]. The goal is to provide stakeholders with relevant analyses to anticipate issues and make decisions in domains with complex multivariate data. For example, a study by Franklin et al [29] modeled the real-time status of a hospital’s emergency department as a dashboard to support rapid clinical decision-making and improve throughput. However, the growing use of dashboards has raised concerns about the cognitive effort required to extract actionable information, particularly for users with limited analytic skills [30]. Data storytelling has been proposed as a more intuitive way to communicate information that motivates decision-making [31]. Stakeholders, however, determined that maintaining and updating multiple specific decision narratives would be overly complex. Therefore, we chose a dashboard approach for its flexibility and adaptability across various public health scenarios, including surveillance and training. In addition, a key design requirement was the ability to customize the dashboard view and generate reports, which was seen as a more scalable and sustainable solution compared to data storytelling, given the diverse decision-making needs of different stakeholders. The primary users of the PANDEM-2 dashboard are public health officers and medics typically trained in data analysis.

#### Review of Existing Dashboards

Responses to the ongoing pandemic provided opportunities to review public health indicators, visualization approaches used, and the design decisions made by different organizations. To gain insights into these aspects, we conducted a review of publicly available dashboards in April and May 2021, operating under the assumption that these reflected the information needs of public health agencies and could be analyzed without an imposition on end users (Multimedia Appendix 2). This review included dashboards recommended by collaborating partners based on their initial user requirements, as well as publicly accessible COVID-19 projects under development (Multimedia Appendix 3). The review assessed the available dashboards, focusing on their aims, target users, and content. We also collated data sources for each dashboard, along with their breakdowns by population, time, location, granularity, and visualization approaches. This analysis provided insights into how organizations prioritized information and the data views expected by experts. It also highlighted different data interaction models and how data artifacts were aggregated, filtered, modified, and recorded. While the data sources identified during this review were considered for inclusion in the PANDEM-2 database, consortium members’ own data eventually proved to be more suitable, especially as larger organizations and governments began openly sharing their information.

#### Data Visualization Catalog

Capturing and storing visualizations for viewing or referencing during the design process was challenging due to their volume and complexity. Initially, screenshots of each visualization, along with related data, were added to a document (Multimedia Appendix 4). However, as the document grew, this approach became impractical and difficult to reference. To overcome this challenge, a data visualization catalog was created. The catalog was specifically developed for annotating and uploading data visualizations during the design phase of the project. The visualizations became searchable using keywords related to graph types, data types, or indicator types. The catalog served as a directory of existing public health visualization approaches, which the project team could consult during the participatory design process. It is important to note that the Vizualisation Catalogue was used solely as a development tool during the design phase and is not part of the final application.

### User Requirements and Participatory Design

#### Gathering of User Requirements

The design and development of the PANDEM-2 dashboard began with collecting user requirements from members of the project consortium, outlining the necessary functionalities and features to meet users’ needs. Participants represented a wide range of domains, including universities, national public health institutes, the Italian Red Cross, the Austrian Red Cross, the National Institute of Medical Emergency (Portugal), a leading institute for trend and technological analysis, and MedTech health organizations. Each consortium partner appointed a contact person to gather internal requirements and communicate them to the project’s technical team.

These designated representatives participated in initial user forums that were held during the project’s inception, aimed at gathering user requirements from domain experts and capturing their needs and preferences for features in pandemic management software (Multimedia Appendix 5). The group was diverse in age, experience, and gender, with participants ranging from recent postdoctorates to professionals with >30 years of experience, and included software designers, data scientists, epidemiologists, microbiologists, physicians, first responders, and project managers. They also represented a wide geographic spread, including Romania, Italy, Portugal, France, Germany, the Netherlands, Belgium, Sweden, Finland, and Ireland. This diversity ensured a wide range of expertise and perspectives, thus reducing potential design biases.

The requirements gathering process involved 2 workshops held between February and April 2021. During these workshops, users described their ideal dashboard for pandemic management and identified essential visual features for the dashboard. These requirements were then compiled into a comprehensive list and organized into functional units. The participatory design surveys were conducted as part of this process, providing interactive design prototypes and collecting feedback from users on key functionalities (Multimedia Appendix 6).

#### Generation and Analysis of User Stories

User requirements were assigned to different groups within the technical team, who analyzed and converted them into user stories. A user story describes a feature from the end-user’s perspective, detailing how it provides value [32] (eg, “As a policy advisor or health care professional, I want to see cases stratified by age, sex, time, and severity to monitor clusters and track the virus spread over time”).

By exploring user requirements and generating user stories, the technical team identified common needs, consolidated similar requests, and clarified key functionalities for the PANDEM-2 system. Each user story generated focused on a primary indicator query and was linked to the initial requirements for traceability. It defined query resources for user interactions, enabling data filtering, aggregation, new data creation, and record management. In addition, each user story considered the time-related and geographic aspects of the indicator within the application. Multimedia Appendix 7 provides an example of a user story.

#### Prioritization and Refinement of Key Variables and Indicators

To refine and prioritize user requirements for the dashboard, a prioritization process was undertaken. This involved a data availability and prioritization survey, which was conducted with the consortium members using the initial requirements generated in the Gathering of User Requirements section. A matrix was created, mapping all necessary variables and indicators required to fulfill the identified needs. This was followed by an assessment of the priorities and availability of these variables in open and restricted data sources. Users were invited to provide insights into their institution’s data availability, including details about the level of information, reporting periodicity, data format, and the geographic level at which the data were accessible (municipal, regional, or national). The questionnaire covered various aspects, such as level of priority, data availability within their institution, the level of detail of publicly available data, adherence to the European Surveillance System (TESSy) format, and the pathogens for which data were collected (Multimedia Appendix 8).

The list of requirements was further divided into 14 variable categories, and each item on the list was classified as either an observation, a characteristic, an indicator, a resource, a document, or a referential. These terminologies are defined in Textbox 1.

**Definitions of variable categories used in the PANDEM-2 dashboard.**

- Observation: Any variable or item that reflects a measurement or observation reported by a data source. These variables are typically numeric and represent concepts like the number of people or resources, or could include nonnumeric items, such as statements (eg, tweets) published by individuals.
- Indicator: Specific observations used to monitor particular questions. They can be categorized as either calculated values with associated methodologies and formulas or directly reported by users.
- Characteristic: Any variable or item which describes or categorizes an observation. It is always linked to variables of the observation type, such as the country, date, or variant of the observed cases.
- Referential: A specific type of characteristic distinguished by having a unique code that can be mapped to other characteristics. For example, municipalities serve as a referential, each possessing a unique code and linking to a country.
- Resources: A specific type of referential that concerns resources needed to handle pandemics. They play an important role in PANDEM-2 for resource modeling features (eg, nurses per bed or material resources). They are associated with observations like the number of available resources.
- Document: Refers to any generic term for a record or written material.

The methods used for defining key indicators and data sources, as outlined earlier, culminated in a final variables list that encompasses both open data sources and data contributed by partners (Multimedia Appendix 9). Alongside this list, data formats and an ETL process were specified to facilitate seamless integration with PANDEM-Source. To support the ETL process, a collaborative effort led to the development of a Microsoft Excel template to accommodate specific data needs for most consortium members. Furthermore, relevant heterogeneous data sources for effective pandemic management, such as Next Generation Sequencing–Laboratory data, social media data, and participatory surveillance data, were identified and corresponding ETL processes were devised for inclusion in the PANDEM-2 database.

**Figure 3:**
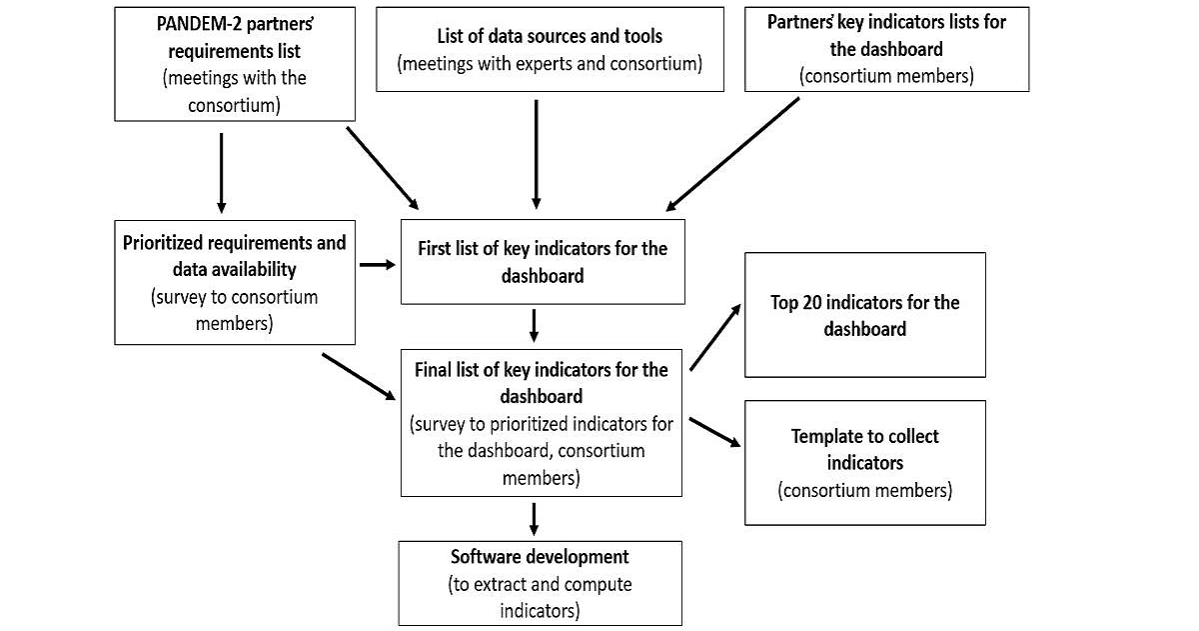
Process used to gather partners' requirements list and prioritize partners' key indicator lists for developing the dashboard’s core features.

After receiving the initial list of requirements and the prioritization feedback, the technical team began further refinement of the requirements. The process was guided by the concept of a minimal viable product (MVP), which focuses on prioritizing essential features to maximize learning with minimal effort [33]. Although the PANDEM-2 system is a prototype, the MVP approach helped in determining where efforts should be concentrated and which requirements should be given priority during the implementation process. Each group in the technical team looked at the requirements list and the prioritization process and created an MVP list. Subsequently, with participation from all Public Health Agencies and first responder partners in the biweekly web-based workshops, the initial requirements, the prioritization list, and the MVPs were combined and deliberated upon. The process of gathering and refining user requirements is illustrated in Figure 3. This process underpinned the development of the PANDEM-Source software used to extract and compute indicators, which were visualized on the PANDEM-2 dashboard. At the end of this prioritization process, a list of key indicators was generated.

#### Prototype Design, Participatory Feedback, and Iterative Improvements

**Figure 4:**
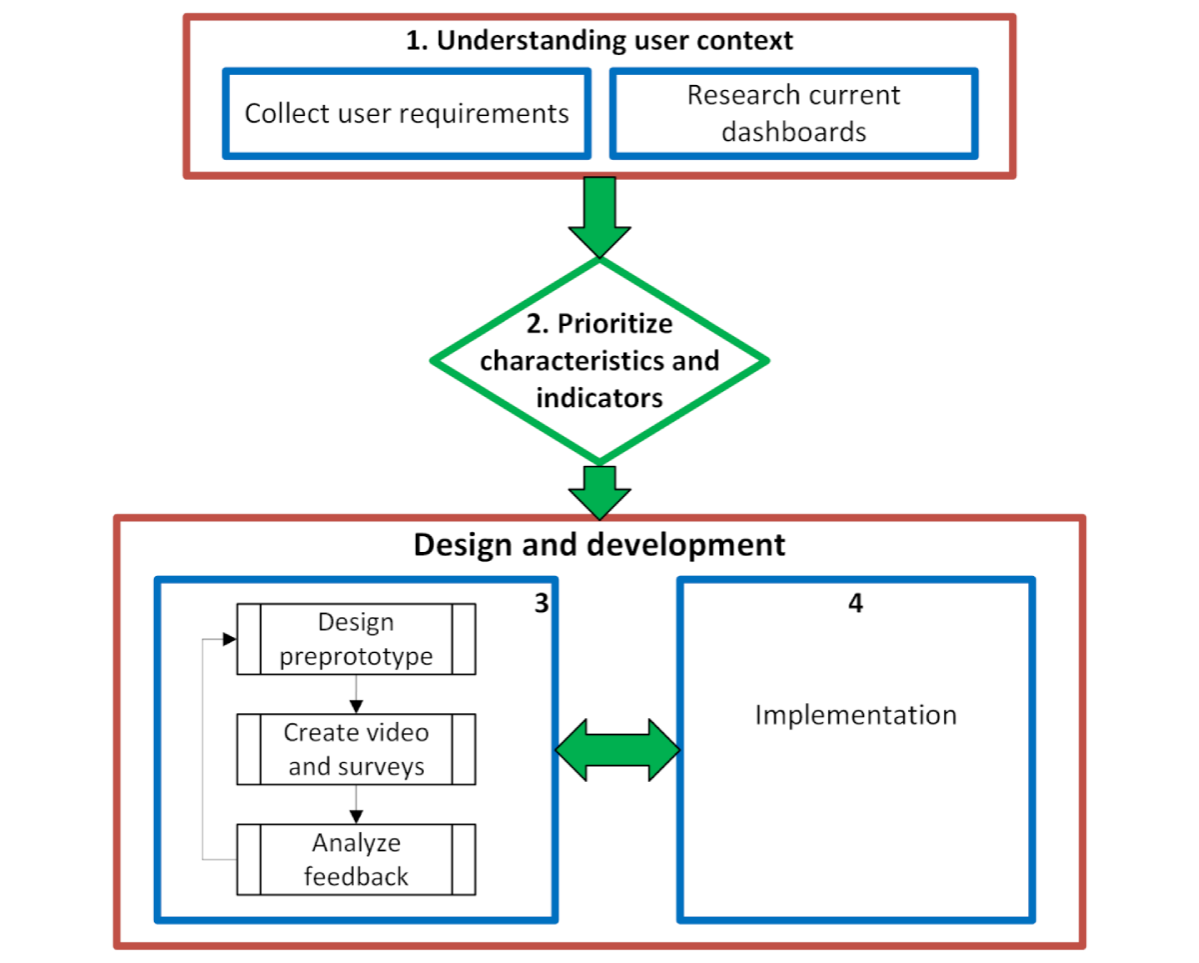
Development workflow, including asynchronous participatory design, detailing the iterative feedback process used to incorporate user input into the PANDEM-2 dashboard design. AVG: average.

The design of the PANDEM-2 dashboard followed an iterative process involving a participatory design approach, with user feedback incorporated throughout the design stages. Participatory design represents a design approach in which the people destined to use the system being designed play a critical role in its design [34]. Generally, participatory design is conducted in face-to-face settings where users are engaged in recording requirements and teasing out functionalities that are necessary to implement them. However, due to the COVID-19 pandemic, these physical meetings were not possible to organize. The participatory design process was therefore adapted to the pandemic context and involved several asynchronous methods, as illustrated in Figure 4. The adapted process allowed relevant stakeholders to participate and enabled collaboration across multiple teams. The analytical components of the dashboard were then designed based on the outcomes of this collaboration and are presented as follows:

1. Understanding the context of use by gathering insights from various stakeholders to understand end-user requirements and identify COVID-19 public dashboards for analysis
2. Prioritization of dashboard characteristics and indicators
3. Asynchronous participatory design (APD) process that involved weekly asynchronous interactions with users. Interactive design prototypes along with video documentation and feedback forms were sent to consortium members, who explored the designs and provided feedback. The primary objective of the APD process was to enable users to interact with a preprototype platform, explore potential interface designs, and provide feedback. This process involved targeted questionnaires, which aimed to explore what graphical representations and interactions were beneficial to users for their respective roles. Participants were presented with a range of potential functionalities linked to priority indicators, which included visual analytics components and interaction models, such as the ability to filter and switch between different data views. Participants shared their feedback on the structure of the pages, the data presented, the data visualizations that were used on each page, and the interactions with those data visualizations. Through this process, users provided feedback on elements to be added, removed, or modified within different components in the system. This feedback was then integrated into subsequent designs.
4. In parallel to the above step, the design prototypes were sent to the software team, who carried out the implementation of designs and improvements toward the final prototype.

#### Impact Assessment

The implementation of ethical, privacy, data protection, and social principles was an integral part of the PANDEM-2 project. The process was led by Trilateral Research, a research and innovation organization specializing in applied ethics, privacy, and data protection in technology development. The project team actively engaged with end users both within and outside the project consortium to identify and mitigate potential risks arising from the tool’s development. Following relevant guidance, including the Information Commissioner’s Office Privacy Impact Assessment Code of Practice [35] and International Organization for Standardization and International Electrotechnical Commission standard for privacy impact assessments [36], the process mapped information flows, assessed potential risks within the system, and provided recommendations to mitigate the risks. A diverse group of stakeholders were involved in the process, participating in 2 workshops conducted in November 2021 and May 2023. There were 20 participants in each of the workshops, representing a wide geographic spread, including Romania, Italy, Portugal, France, Germany, the Netherlands, Belgium, Sweden, Finland, and Ireland. Those that partook in the workshops came from universities (UCLouvain and University of Galway), national public health institutes (Institute of Public Health in Romania, Public Health Agency of Sweden, the National Institute for Public Health and the Environment in the Netherlands, the Robert Koch Institute, the Portuguese National Emergency Medicine Institute, and the Finnish Institute for Health and Welfare), as well as other organizations such as the Italian and Austrian Red Cross, the Fraunhofer Institute for Technological Trend Analysis, and software companies specializing in health, Epiconcept, and development, Clarisoft. Roles of those taking part included software design and development, data science, epidemiology, microbiology, infectious disease specialists, doctors, researchers, first responders, pandemic managers, and project managers. Notably, participants included a former adviser to the World Health Organization on health security and a former head of the Chemical, Biological, Radiological and Nuclear threat unit for Belgian defense. The initial workshop aimed to raise awareness of ethical, privacy, data protection, and social principles requiring consideration during the development and deployment of the PANDEM-2 system and tools. It also focused on mapping data flows within the PANDEM-2 system and identifying related risks or opportunities. In addition, the workshop explored privacy, social, and ethical issues and opportunities to be considered in different pandemic scenarios, both in the preparedness and response phases. Possible mitigations for the identified risks were also discussed. The second workshop involved an open and collaborative discussion, allowing stakeholders to contribute insights on mitigation measures, necessary actions, and final recommendations for both project stakeholders and end users.

### Ethical Considerations

The PANDEM-2 project adhered to GDPR regulations and Horizon 2020 guidelines. Ethical approvals were obtained from the relevant institutional review boards of all consortium partners involved in data collection and processing. These approvals ensured that the project complied with the highest ethical standards, protecting participants’ rights and ensuring the responsible handling of personal data. Informed consent was obtained from all participants before their involvement in the stakeholder engagement activities. The consent procedures ensured that participants clearly understood the nature of the study, the use of their data, and their rights, including the ability to withdraw at any time. Participants were also informed that their data would be used for research purposes only. All personal data were anonymized or pseudonymized to protect participants’ identities. Data were securely stored on password-protected servers, accessible only to authorized personnel. Participant identifiers were removed or replaced with pseudonyms to ensure anonymity in the research outputs, preventing any risk of reidentification. No financial compensation was provided to participants. Participation was entirely voluntary, and participants had the right to withdraw from the study at any time without any negative consequences. No identifiable images of participants are included in the study materials or outputs.

## Results

### System Architecture and Data Integration

#### Implementation and Configuration

The dashboard was implemented and hosted on the Amazon Cloud as a central data management system at the EU level, accessible to all national health institutes across Europe. To address challenges with semitransparent data access, whereby some but not all data from different institutions can be shared with others, the architecture is not only open access but also adaptable. An adaptable architecture allows for both isolation of data between groups of countries and on-demand installation or deployment in a single health institute.

The dashboard offers flexibility in its configuration, allowing customization in 2 main areas. First, user experience and user-interface attributes, which encompass elements related to user experience and user interface. This involves tailoring aspects such as the language used for interface elements, the design of graphical features, and the overall visual style. Users can personalize the appearance of the dashboard to align with their preferences and needs by adjusting these attributes. Second, internal functional parameters that deal with the technical aspects of the dashboard’s operation can be customized. This includes details on how different modules within the system access APIs and specific technical information relevant to the system’s functioning. The administration module of PANDEM-2 serves as the control center for managing these parameters, granting users the ability to fine-tune the dashboard’s functions and adapt it to their specific requirements.

#### Data Sources and Key Indicators

**Figure 5:**
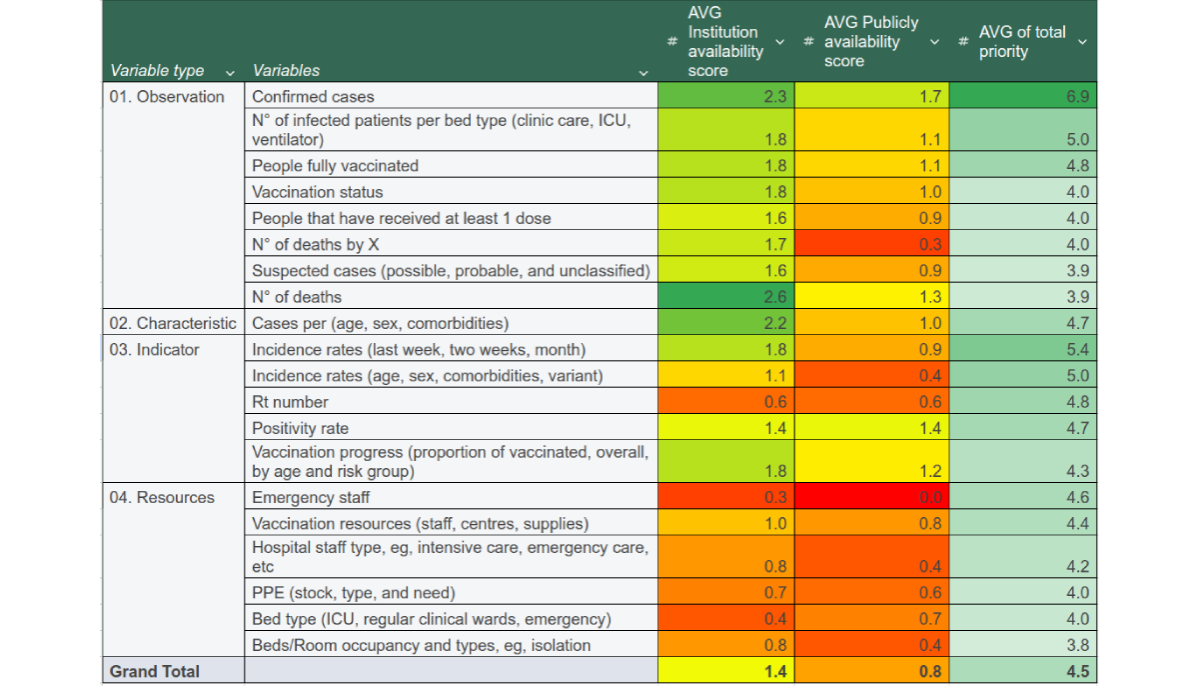
Survey results highlighting the availability and prioritization of key data variables for the dashboard (AVG; X). AVG: average; ICU: intensive care unit; N°: number of; PPE: personal protective equipment; Rt:; X: specific pathogen of the pandemic.

**Figure 6:**
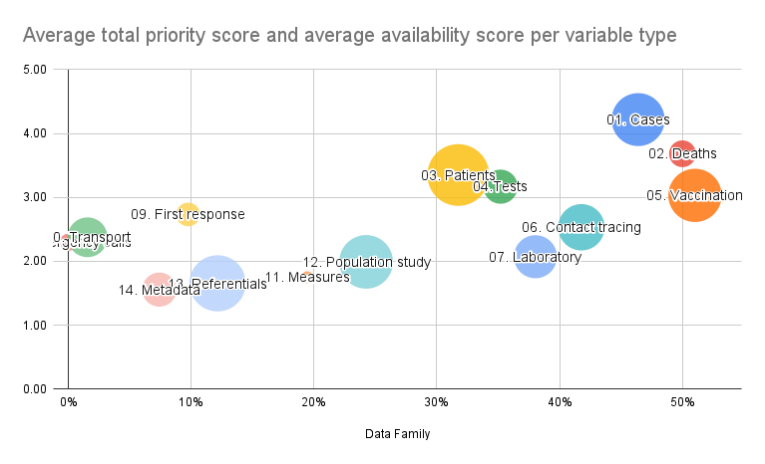
Comparison of priority versus availability of variable categories, showing the alignment between prioritized variables and their availability across institutions.

Responses to the data availability and priority survey are shown in Figures 5 and 6, and tables 1 and 2. The 9 participant institutions were questioned about 103 variables classified by groups. For each variable, the respondent answered a series of questions about availability, priority, and format of the associated information. On the basis of these answers, data availability scores were calculated and color-coded, ranging from low (red) to high (green; Figure 5). The variables identified as most relevant were cases, vaccination uptake, deaths, hospital resources, and hospital admissions. Figure 6 shows that the most important variable categories were also the categories with the most available data. Higher priority categories are toward the top of the graph, while high availability categories are toward the right. This observation highlights the importance of prioritizing and ensuring accessibility to critical data categories to support effective pandemic preparedness and response efforts.

Most of the data were reported on a weekly or daily basis, indicating a commitment to timely and up-to-date information (Table 1). A significant proportion of the data were collected and recorded by various institutions and from data sources related to COVID-19. There was limited recording of pandemic-related data before the COVID-19 pandemic. There were also variations in data granularity, reporting frequency, geographic coverage, or other specific details related to the COVID-19 information being collected. On average, only 9.7% (10/103) variables were available in the TESSy [37] format and 26.2% (27/103) only in a different format, a format used by the TESSy (Multimedia Appendix 10).

**Table 1:** Data reporting frequency and types of pandemic-related data available, highlighting the frequency of reporting (daily, weekly, or monthly) and the prevalence of COVID-19 data from various sources (n=115).

Data category			Reporting variables or items, n (%)
**Periodicity**			
	Weekly	17 (14.7)	
	Daily	14 (12.1)	
	Others^a^ (specify in comments)	8 (6.9)	
	Monthly	2 (1.7)	
**Pathogens**			
	COVID-19	17 (14.7)	
	Others (specify in comments)	13 (11.3)	
	Influenza and COVID-19	8 (6.9)	
	Influenza	1 (0.8)	

^a^The “others” option refers to any reporting frequencies that were neither daily, weekly, nor monthly, which participants were asked to provide qualitatively through comments.

The survey also identified several data gaps, as shown in Table 2. Among these, 2 areas of particular significance were contact tracing and deaths. It was evident that these areas lacked sufficient information despite their high priority in the survey. In addition, there was also a noticeable lack of information concerning resource availability, despite its recognized importance in pandemic management.

**Table 2:** Data gaps identified during data availability survey, showing critical pandemic-related data, such as contact tracing and deaths that were insufficiently available despite high priority.

Variable category	Average public availability gap
Deaths	1.1
Contact tracing	0.9
Cases	0.8
Laboratory	0.6
Patients	0.5
Vaccination	0.5
Tests	0.3
Referential	0.2
Population study	0.1
Metadata	0.1
First response	0.1
Transport	0.0
Emergency calls	0.0
Measures	0.0

The public availability gap is a metric quantifying the specific types of data accessible at a detailed level but not disclosed publicly. Each participant provided their estimation of variable availability within their institution and at the public level, to the best of their knowledge. The assigned scale attributes 4 as the highest value to individual level, 3 to municipality, 2 to regional, and 1 to country level. In instances where data were unavailable or unknown, a score of 0 was assigned. The data availability gap is determined by calculating the difference between the average availability score at the institution level and at the public level.

**Figure 7:**
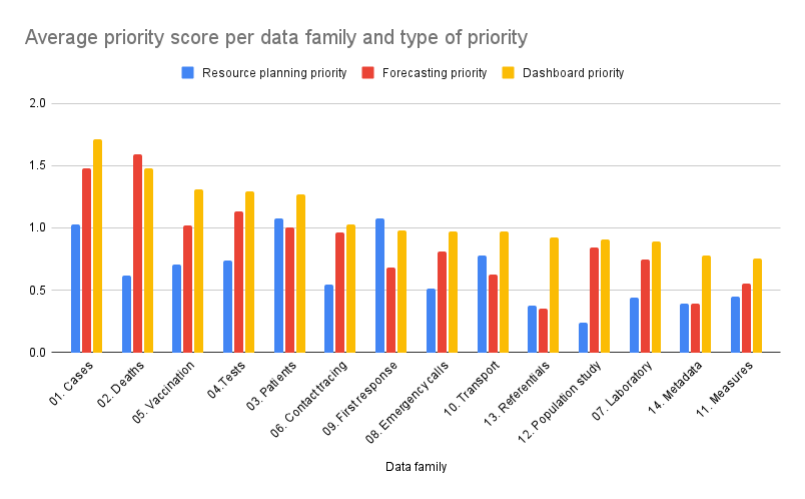
Prioritization of variable categories for resource planning, forecasting, and the dashboard, showing importance ratings assigned to various data categories by participants.

Figure 7 illustrates the findings regarding the variables that users prioritized for resource planning, forecasting, and the dashboard. To calculate the priority score for these purposes, participants were asked to rate the importance of each variable from 0 to 3 for the 3 purposes, with 0 being the lowest and 3 being the highest. The results were averaged by variable category. In most cases, dashboard variables held a higher level of importance compared to forecasting or resource planning variables. The spikes observed in the resource planning and forecasting areas highlight the data points of utmost priority for those domains, with particular significance placed on the ability to forecast deaths and to perform resource planning for patient care at the hospital and first response level.

At the end of the data and indicator prioritization process, a template to collect variables and indicators from nonopen sources (or partner-restricted data) was created.

### Dashboard Design and User Engagement

#### Insights and Outcomes From Dashboard Review

The review of existing dashboards provided several insights that influenced the design of the PANDEM-2 dashboard. The data visualizations within the reviewed dashboards highlighted the prioritization of information by various organizations and identified the data views expected by experts. This review also revealed different data interaction models useful in public health domains and how data artifacts were aggregated, filtered, changed, and recorded. Insights gained from this review helped shape the decision to use consortium members’ data as the primary data source, as these proved to be more comprehensive and reliable than many external sources. The review also informed the design features and visualization approaches adopted in the PANDEM-2 dashboard.

#### Prototype Design and Features

Multimedia Appendix 11 visually illustrates the design of a PANDEM-2 dashboard page. The key components on each page of the dashboard include the following:

1. Overview explanation component. This component includes a page title and provides an explanation of the indicator and the data presented on the page. It serves as a brief summary to provide context and understanding.
2. Indicator cards. These cards display high-priority data related to the indicator. They offer a quick snapshot of the current state of the pandemic, allowing users to grasp important information at a glance. The cards also feature an information button that provides additional details about the data being shown. The highlighted figures show a comparison between the most recent 7 days and the preceding 7 days. The highlighting provides an instant visual understanding of how important indicator variables such as admissions and intensive care unit admissions are changing over time.
3. Map. The map component offers a geographical perspective on the pandemic situation. Users can choose the specific information they wish to view on the map, such as cases, vaccination rates, or other relevant metrics. They can also segment the map by NUTS regions, providing a more localized view.
4. Graphs. The dashboard includes multiple graphs that present detailed information about the indicator. These graphs allow users to explore and filter the data according to their preferences. This feature enables a deeper analysis of the pandemic trends and patterns.
5. User-interface functionality. Users can access the PANDEM-2 system using the interface in the presentation layer. This interface offers multiple possibilities to display and filter information, build reports based on displayed graphical elements and comparisons between different data, and export these reports in many predefined formats, including images, tables, and web service-specific formats.

The final design framework of the PANDEM-2 dashboard was structured to provide an intuitive and interactive experience for users. Each indicator view was conceptualized as a dedicated page, comprising various relevant components. Abstract components were developed that could be adapted to each indicator page, ensuring a unified design and interaction experience across pages. This approach allowed users to intuitively understand the interactions and functionality across different pages once they had become familiar with the system on one page.

Together, these components enhance the accessibility and usability of the PANDEM-2 dashboard, empowering users to gain insights into the pandemic data through both high-level summaries and detailed visualizations. More illustrations of initial and final dashboard designs are shown in Multimedia Appendix 12.

### Participatory Design Feedback

The participatory design process led to several modifications and improvements in the PANDEM-2 dashboard prototype. The number of responses to each of the APD feedback forms ranged from 4 to 9.

Feedback from the APD process highlighted the importance of data visualization in diverse formats, combining different data types, and analyzing data across various time frames. Users expressed their interest in generating custom reports and exploring data alongside government interventions (countermeasures):

The addition of countermeasures on the emotion/sentiment timelines is good. However, the countermeasures would be easier to see or understand, if they were all on the top part of the graph with the name of each intervention placed next to the line [on the left or right side of the graph]. Now it’s difficult to check back to the figure legend below and tell one shade of grey from another. Now there’s also a false impression that a countermeasure is somehow linked to the emotions on the same level of the graph

for example, social distancing with anticipation and disgust

Because of the feedback received, the final prototype incorporated various features that catered to the specific needs of the stakeholders, such as customizable dashboard views and report generation capabilities. The design also addressed the cognitive effort required to extract actionable information, ensuring that the dashboard remained user-friendly and accessible to all users, regardless of their analytical skills. This feedback proved invaluable for the technical team, offering insights into the preferred indicators and formats for their presentation. In addition, the feedback offered the technical team an understanding of how end users intended to use the data beyond the dashboard, including saving reports, downloading visualizations, and obtaining underlying data. When asked about saving outputs from the dashboard, participants expressed a desire for flexibility, with various preferences emerging. Users wished to save the current state of the page to share with others, download images or PDFs of the page, obtain the data within the page, or capture images of specific sections of the page.

Regarding epidemiological and resource modeling, participants expressed the desire to save the model used, the input parameters for scenario runs, visual outputs, data outputs in alternative formats, and high-level indicators.

Participants’ feedback also shed light on data reporting challenges, particularly in terms of disparities in reporting levels and time intervals and the need for standardization across organizations. For instance, a participant highlighted how delays in testing workflows in Finland resulted in challenges when reporting cases and tests:

Due to unavoidable delays in testing workflow [sampling, sample shipping and processing, analysis, registration of results] it may not be realistic to report tests and cases ‘this week.’ In reality [in Finland], numbers of cases and tests from this week cannot be reported before Wednesday [COVID-19] or Friday [other viruses in infectious diseases register] next week. Sentinel surveillance may have even longer delays.

The APD process further brought to light the complexity of data in public health situations, emphasizing the significance of considering interdependencies. It highlights that the system developed serves as a decision support tool and that the role of public health officials remains crucial in making informed decisions based on the presented information. One participant’s feedback from Germany highlighted a specific example of complexity in data representation related to counting beds versus assessing capacity:

In Germany we do not really count the beds but the capacity. This is because to be able to add a new patient you need a bed, a room, ventilation, staff etc, or do you mean capacities with the term “bed”?

This inquiry highlights the importance of clarity and precision in defining data elements to ensure accurate interpretation and decision-making within the tool. Finally, through their engagement with an expanding collection of interactive designs, users were able to identify gaps in the available indicators and provide specific requests for additional information, such as excess mortality data. This iterative process was instrumental in ensuring continuous and active user involvement throughout the project, enabling their valuable contributions to the design and development of the PANDEM-2 system.

### Impact Assessment Findings and Mitigation Strategies

The impact assessment process identified 10 potential risks, all of which were deemed to have a low to medium likelihood of occurring. In total, 4 (40%) of the risks were ethical, 2 (20%) regarded privacy and data protection, and 4 (40%) related to social factors. These are summarized in Textbox 2 and reported in more detail elsewhere [38].

**Summary of the identified risks categorized into ethical, privacy and data protection, and social factors.**

**Ethical risks**

- Accessibility and inclusivity: risk of excluding individuals with certain needs.
- User autonomy: risk of limiting end-users’ control over their use of the training platform.
- Responsible use: risk of incorrect or unethical use of the training platform.
- Transparency and accountability: risks associated with the ability to account for actions taken or decisions made during the development of the training platform.

**Privacy and data protection risks**

- Security and access controls: risks relating to the protection of digital information from unwanted actions or unauthorized users, based on commonly accepted security frameworks, such as the confidentiality, integrity, and availability model.
- Data reidentification: risk associated with the correlation or linking of anonymous datasets with other data sources—especially those that have been collected by the state

**Social risks**

- Unintentional bias: risks associated with biased algorithms, biased data sources, and bias in training content that may lead to systematic errors that create prejudiced or unintended outcomes.
- Data quality and accuracy: risks associated with the ability to make informed decisions based on accurate information.
- Dependency on technology: risk of heavy reliance on the IT platform for critical operations, processes, or decision-making.
- Collaborative development: risk of inadequate collaboration with end users or public health authorities in the development and postproject stages to understand training needs and enhance the platform’s capabilities.

The risks were effectively mitigated through several measures throughout the project. Design considerations extended beyond functionality to inclusivity and accessibility. The user interface was designed to accommodate multilingual users, providing options in both English and Romanian with provisions for translation into additional languages. Accessibility for individuals who are visually impaired was ensured through the selection of a color-blind–friendly palette. To facilitate ease of use, user-friendly training materials and guides were developed, offering clear instructions for system navigation. Supplementary features such as tooltips and contextual help were integrated, further enhancing the user experience. In addition, transparency was prioritized through the provision of accessible data sources and modeling assumptions used in the development process. Security was a paramount concern, prompting the adoption of Amazon Cloud, a reputable cloud computing service known for its robust security measures. The commitment to open collaboration and adaptability was evident in the decision to offer all components of the dashboard as open-source software. This approach encourages wider use, fosters adaptability, and invites collaboration across diverse contexts. Furthermore, a comprehensive developer guide in English was provided, serving as a valuable resource for future developers interested in building upon the PANDEM-2 platform or creating similar tools. End-users’ feedback was continuously obtained and integrated to ensure the platform’s suitability to diverse user needs.

To enhance the accuracy and relevance of training content, public health authorities’ expertise was used in content development, and active end users engaged in patient surveillance and treatment assessed the training materials. This collaborative approach guarantees that the training content accurately reflects current information, best practices, and public health guidelines. Training exercises were based on scripts generated by public health authorities, adding objectivity and minimizing subjective biases or arbitrary preferences. These actions collectively enhance the accuracy and scientific validity of the training content.

## Discussion

### Principal Findings

The PANDEM-2 project aimed to develop an IT prototype system that offers a standardized and interactive platform for pandemic preparedness training and response to support decision-making on pandemic management. This paper outlined the development and potential of the PANDEM-2 dashboard, including methodologies used to gather users’ insights to enhance its design and steps taken to identify and address potential risks associated with the technology.

The COVID-19 pandemic brought about an unprecedented surge in pandemic-related data at national, EU, and global levels, presenting a valuable opportunity to identify crucial data needs for effective pandemic response. PANDEM-2 leveraged user surveys to identify essential data requirements, not only for the ongoing pandemic but also for potential future health threats. Despite this increase in data, significant challenges emerged due to data availability, diverse data sources, nonstandardized formats, varying granularity, and data sharing restrictions among EU member states. These obstacles hindered timely data exchange for a coordinated EU-wide response and hampered cross-border response efforts. Addressing these data challenges became a central concern throughout the PANDEM-2 project, and consequently it emerged that it is imperative to proactively anticipate and address data requirements for potential future pandemics.

The EU initiated measures to address data sharing gaps through Regulation 2022/2731 on serious cross-border threats to health [39]. The Commission’s responsibility includes defining mechanisms for data exchange, adhering to personal data protection rules and information exchange security. In addition, Article 5 of Regulation 2022/2730 on serious cross-border threats to health specifically refers to the development of the Union health crisis and pandemic plan “to promote effective and coordinated response to cross-border health threats at Union level” [40]. Specifically, article 5, point 5, states, “In order to ensure the operation of the Union preparedness and response plan, the Commission shall conduct stress tests, exercises and in-action and after-action reviews with Member States and update the plan as necessary.”

The development process of PANDEM-2 aligns with these measures, both in terms of data sharing and exercises. Lessons learned in the development of the platform offer insights into data analysis, sharing, and reporting methods, effectively supporting public health actions and control measures. The PANDEM-2 platform’s design, built on an open architecture and open data sources, ensures security, stability, extensibility, and flexibility, allowing it to adapt to evolving data needs and data sharing practices.

The PANDEM-2 platform is also a training platform, designed to facilitate both national and cross-border simulation exercises. A simulation exercise is a form of training or evaluation of capabilities involving the simulation of an emergency [41]. Operationally, there is the potential for the PANDEM-2 dashboard to be deployed at regional, subnational, national, and EU levels to conduct simulation exercises aimed at stress-testing pandemic response capacity. A recent large-scale functional exercise involving 2 public health emergency operating centers within the PANDEM-2 consortium (the Robert Koch Institute in Germany and the National Institute for Public Health and the Environment in the Netherlands) supports this position. The aim of this exercise was to explore the coordinated response to a large-scale pandemic due to a novel strain of avian influenza in Europe with the objective of testing and evaluating the functionalities of the PANDEM-2 IT system and associated tools. During the 2-day exercise, participants received 44 injects to develop the scenario and provide specific tasks to complete within their country’s context. A manuscript outlining the implementation of this exercise is currently under review [11]. Evaluation of the exercise comprised both qualitative (hot-wash group interviews and after-action interviews) and quantitative (questionnaires and interviews), and feedback indicated that the PANDEM-2 dashboard met or exceeded their technical requirements in terms of user experience. Moreover, participants agreed that the dashboard addresses a current gap in national and cross-border training capacity for pandemic preparedness and response.

Alongside addressing data requirements, effective methods for data presentation need to be considered. The PANDEM-2 platform’s participatory design approach emphasizes the importance of involving end users in the design of a system. Through this interactive and iterative approach, users can define system requirements, share domain expertise, and identify high-priority indicators for pandemic management. This approach within the PANDEM-2 platform enabled the creation of a design framework that offers adaptability and scalability, ensuring consistent interactions even as indicators evolve.

The participatory design approach also supported the effective knowledge transfer among end users and the technical team. This was crucial to enable the design team to understand the requirements of diverse and specialized domains and to facilitate the design of novel technical solutions within those domains. Ongoing research endeavors should be directed toward enhancing user engagement with intricate software in specialized areas, such as epidemiological and resource models, balancing user-friendly interfaces with necessary complexity, and exploring efficient techniques for presenting voluminous datasets and findings.

Data storytelling has become one such technique for enhancing user engagement and is often seen as an intuitive way to drive decision-making and reduce the risk of information overload. The participatory design process revealed that managing multiple decision narratives would add unnecessary complexity. The dashboard approach chosen provides flexibility across public health scenarios like surveillance and training. The ability to customize views and generate reports makes it a more scalable solution, especially for our primary users, public health officers, and medics trained in data analysis, who require more in-depth exploration than storytelling offers. The dashboard includes filtering, tagging, and prioritization features to address information overload, but managing complex data while meeting the needs of expert users remains a challenge. The participatory design process helped refine key indicators to present concise, relevant information, but balancing exploration and clarity is an ongoing effort in systems like PANDEM-2.

The identification of high-priority indicators and the possibility of consolidating them into a singular variable that captures the status of a pandemic merits in-depth investigation. This strategy could streamline decision-making processes and yield immediate insights into the dynamic nature of the pandemic across its various stages.

### Summary

During the project, challenges related to data standardization across consortium members became evident, particularly due to the lack of data in the TESSy format. In addition, many countries had regional divisions that differed from those used by the ECDC (NUTS). End-user experts also highlighted the critical role dashboards play in pandemic preparedness and response. The findings underscore the importance of making data for these indicators accessible for public health purposes.

The project’s commitment to responsible innovation and user-centric principles was demonstrated through its robust impact assessment process, which prioritized key elements such as privacy-by-design and adherence to GDPR as well as regulations governing health technology development. This iterative process ensured that the platform remained relevant and user-friendly. Transparency stands as a core value of the PANDEM-2 project, demonstrating its commitment to openness and accountability. All project resources have been made publicly available for interested parties to access and review on the project website [42] and the European research platform Cordis [43], where stakeholders can find comprehensive information about data sources used, project procedures, and methods.

While the PANDEM-2 dashboard holds promise for effectively managing real-time data during future pandemics, successful integration with existing IT systems within public health agencies will be a necessary step. In the interim, the dashboard stands as an innovative training platform, providing member states the opportunity to assess their pandemic preparedness capacity through collaborative cross-border exercises developed by the PANDEM-2 project. Despite the limitations encountered during the project, our approach and findings provide valuable insights through open-access materials and tools to support public health agencies in EU member states to better prepare for upcoming pandemics.

To ensure comprehensive and transparent reporting of the development process, this study followed the Guidelines for Intervention Development checklist, documenting each stage of the dashboard’s design and stakeholder engagement (Multimedia Appendix 13) [44].

## Data Availability

The data sets generated and analyzed during this study are available from the corresponding author on reasonable request.

## Appendix

Multimedia Appendix 1 Security and data protection aspects of the dashboard.

Multimedia Appendix 2 Dashboards reviewed.

Multimedia Appendix 3 List of COVID-19–related projects.

Multimedia Appendix 4 Example of data visualization entered in document.

Multimedia Appendix 5 Checklist for reporting results of the initial user requirements survey.

Multimedia Appendix 6 Checklist for reporting results of internet e-surveys: participatory design.

Multimedia Appendix 7 Example user stories.

Multimedia Appendix 8 Data availability and prioritization survey questions.

Multimedia Appendix 9 Final list of variables by variable category and requirement type.

Multimedia Appendix 10 Existing data in the European Surveillance System format.

Multimedia Appendix 11 Example design of dashboard page illustrating key components: (1) explanation header; (2) indicator card; (3) map; and (4) graph. NUTS: Nomenclature of Territorial Units for statistics (a hierarchical system for dividing up the economic territory of the European Union). Implemented dashboard page in the PANDEM-2 prototype, reflecting design feedback with features like customizable reports and enhanced data visualization.

Multimedia Appendix 12 Illustrations of initial and final dashboard designs: progression of the map component.

Multimedia Appendix 13 Guidelines for Intervention Development checklist.

## Abbreviations

APD: asynchronous participatory design
API: application programming interface
AWS: Amazon Web Services
ECDC: European Centre for Disease Prevention and Control
ETL: extract, transform, and load
EU: European Union
GDPR: General Data Protection Regulation
MVP: minimal viable product
NUTS: Nomenclature of Territorial Units for Statistics
TESSy: European Surveillance System
WHO: World Health Organization
