# Supplementary material for: Building and Developing a Tool (PANDEM-2 Dashboard) to Strengthen Pandemic Management: Participatory Design Study
Source: JMIR Public Health Surveill. 2025 Mar 5;11:e52119. doi: 10.2196/52119 (PMC11923449; doi:10.2196/52119)
Supplement: Multimedia Appendix 1 [file publichealth_v11i1e52119_app1.docx]

| DATA PROTECTION |  |
| --- | --- |
| *Protecting Data during transport* | The PANDEM-2 API provides HTTPS endpoints using TLS for communication, thus providing encryption in transit during communication. |
| *Enforce encryption at rest.* | We ensure that the only way to store data is by using encryption.   1. Encrypted volumes on all servers 2. Encrypted backups |
| *Code review for OWASP best practices* | All code is reviewed before going into production and reviews are focused on security like avoiding injection flaws, broken authentication or access control, cross-site scripting, etc. |
| *Use only verified libraries without security issues.* | All libraries are verified with npm before use. Also, we use only libraries with a very wide user base and support (including regular updates) and do not make unplanned changes to the libraries:  1) Automated code deployment using continuous integration and delivery.  2) Inherited security from development frameworks Angular and MongoDB.  3) Identity & access management |
| *Authenticate network communications* | PANDEM-2 is using network protocols that support authentication and allow for trust to be established between the parties. This adds to the encryption used in the protocol to reduce the risk of communications being altered or intercepted. |
| *Limit access to the network: Access to the network is limited to* | a) HTTPS access to the front web servers, while the database is not directly accessible to the public.  b) Access to databases is limited to the application tier that uses the database on specific ports using network security groups.  c) Administrative connections to the infrastructure are restricted to a set of known IP addresses and require the use of a key.  d) IAM Role-based access control for infrastructure different from the root account. |
| *Data access:* | Data access is limited to a minimum amount for each role, and all changes are logged.   1. Use the JWT internet standard. 2. Enforce role-based access control on all requests. |
| *Data protection* | AWS Key Management Service (KMS) manages keys used for data encryption at rest.  AWS Secrets Manager stores and controls access to credentials like database credentials outside, further increasing the security of data.  Security certificates are provisioned, managed, and deployed using AWS Certificate Manager. |
|  |  |
| INFRASTRUCTURE |  |
| *Infrastructure protection* | We protect the platform by filtering traffic based on rules that filter web requests based on IP addresses, ports, and HTTP headers. |
|  | |
| **THREAT DETECTION & CONTINUOUS MONITORING** | |
| *Threat detection* | Amazon GuardDuty continuously monitors for malicious activity and unauthorised behaviour to AWS accounts, workloads, and data stored in Amazon S3. |
| *Secure & separate storage for passwords using AWS Secret Manager* | We have implemented secure storage for passwords using AWS Secret Manager |
| *Infrastructure monitoring* | Monitor activity in the AWS account using Cloud Watch and Cloud Trail. |
