# Supplementary material for: Building and Developing a Tool (PANDEM-2 Dashboard) to Strengthen Pandemic Management: Participatory Design Study
Source: JMIR Public Health Surveill. 2025 Mar 5;11:e52119. doi: 10.2196/52119 (PMC11923449; doi:10.2196/52119)
Supplement: Multimedia Appendix 4 [file publichealth_v11i1e52119_app4.pdf]

France covid dashboard

Link: [CovidExplorer Explorez les données Covid19 en France]

Description: It is a tool for exploring data from Covid-19 in France. It offers three modules: Territories, Age groups and Datatypes.

It allows to:

- Change y-axis by selecting epidemic indicator(e.g., incidence rate,positive cases), health indicator (e.g.,hospital admission,hospital deaths) or vaccination indicator(e.g,number of people vaccinated)
- Compare several territories by selecting an epidemic indicator, health indicator or vaccination indicator
- Analyse the pandemic impact in age groups in a region by an epidemic indicator, or health indicator
- explore the evolution of the pandemic in a region by type of data (e.g., incidence rate, hospital admission, positive rate)

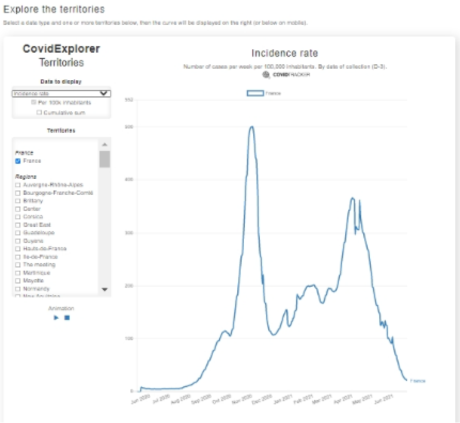

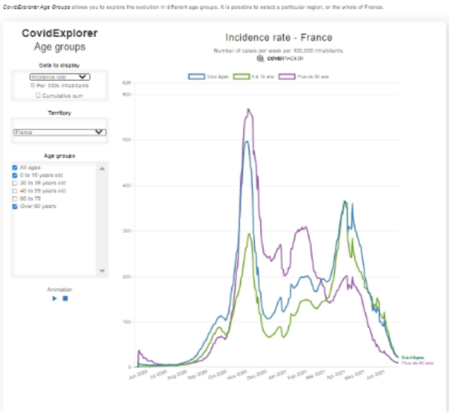

.
