# Supplementary material for: Building and Developing a Tool (PANDEM-2 Dashboard) to Strengthen Pandemic Management: Participatory Design Study
Source: JMIR Public Health Surveill. 2025 Mar 5;11:e52119. doi: 10.2196/52119 (PMC11923449; doi:10.2196/52119)
Supplement: Multimedia Appendix 5 [file publichealth_v11i1e52119_app5.docx]

| **Checklist for Reporting Results of Internet E-Surveys: Participatory Design** | | |
| --- | --- | --- |
| All participatory design surveys followed the same format. The responses in the checklist below are the same for each survey | | |
| **Item Category** | **Checklist Item** | **Explanation** |
| Design | Describe Survey Design | Users from 9 respondent institutions went through a process of user requirements gathering on dashboard, forecasting and modelling news for pandemic management. A list of 103 initial variables were extracted from user requirements and then each user has to answer eight questions from each variable. Questions included subjects about data availability, priority and format of available data at their institution. The questionnaire was sent as an excel file which was fulfilled and returned by each participant. |
| IRB (Institutional Review Board) approval and informed consent process | IRB Approval | NA |
|  | Informed Consent | The subjects were informed of the purpose of the survey, anonymity, confidentiality, and voluntary principles before responding. |
|  | Data protection | The questionnaire was sent to specific respondents. No additional personal information was gathered. The collected data was stored in a shared google drive with those on the project, including the respondents. Only specific personnel within the project can view and analyse the data |
| Development and pretesting | Development and testing | Respondents' data was collected via email. Respondents were asked about data availability, priority and format of available data at their institutions. |
| Recruitment process and description of the sample having access to the questionnaire | Open survey versus closed survey | Closed survey aimed at potential users of the dashboard |
|  | Contact mode | Email |
|  | Advertising the survey | Closed survey directed at specific respondents so no advertisement of the survey was done |
| Survey Administration | Web/E-mail | The survey was emailed to respondents as an attached excel file. |
|  | context | A google drive folder was used for the project to collect and aggregate data |
|  | Mandatory/Voluntary | Surveys were Voluntary |
|  | Incentives | Respondents were possible end users of the developed application |
|  | Time/Date | The data was collected between August 2021 and October 2021 |
|  | Randomization of items or questionnaires | Questions were not randomised |
|  | Adaptive questioning | None of the questions were required. |
|  | Number of Items | Eight questions for 103 identified elements |
|  | Number of Pages | 1 spreadsheet with 103 rows and 8 columns |
|  | Completeness check | None. This was a voluntary survey with no required questions. |
|  | Review step | Performed by each respondent avant submission. |
| Response Rates | Unique site visitor | N/A |
|  | View rate (Ratio of unique survey visitors/unique site visitors) | N/A |
|  | Participation rate (Ratio of unique visitors who agreed to participate/unique first survey page visitors) | The survey was sent to 11 institutions and we got 9 surveys returned. |
|  | Completion rate (Ratio of users who finished the survey/users who agreed to participate) | Completion rate cannot be calculated since questions were not mandatory. |
| Preventing multiple entries from the same individual | Cookies Used | None |
|  | IP check | None |
|  | Log File Analysis | N/A |
|  | Registration | N/A |
| Analysis | Handling of incomplete questionnaires | All surveys were analysed. No questions were mandatory. All feedback was considered |
|  | Questionnaires submitted with an atypical timestamp | Response data had a timeline. Surveys submitted after that time were considered where appropriate at a later date. |
|  | Statistical Correlation | N/A |
