# Supplementary material for: Building and Developing a Tool (PANDEM-2 Dashboard) to Strengthen Pandemic Management: Participatory Design Study
Source: JMIR Public Health Surveill. 2025 Mar 5;11:e52119. doi: 10.2196/52119 (PMC11923449; doi:10.2196/52119)
Supplement: Multimedia Appendix 6 [file publichealth_v11i1e52119_app6.docx]

| **Checklist for Reporting Results of Internet E-Surveys: Participatory Design** | | |
| --- | --- | --- |
| All participatory design surveys followed the same format. The responses in the checklist below are the same for each survey | | |
| **Item Category** | **Checklist Item** | **Explanation** |
| Design | Describe Survey Design | The participatory design surveys were accompanied by an interactive design of the planned page and a video demonstration of the page and its functionalities. Users were to watch the video, use the interactive demo and then answer specific questions about the design and functionality. Users were also asked for any additional feedback that they may have had. |
| IRB (Institutional Review Board) approval and informed consent process | IRB Approval | NA |
|  | Informed Consent | The subjects were informed of the purpose of the survey, anonymity, confidentiality, and voluntary principles before responding. |
|  | Data protection | The questionnaire was sent to specific respondents. No additional personal information was gathered. The collected data was stored in a shared google drive with those on the project, including the respondents. Only specific personnel within the project can view and analyse the data |
| Development and pretesting | Development and testing | Respondents’ data were collected via email and google forms. Respondents were asked about functionalities, and components types. Later surveys took into account previous feedback |
| Recruitment process and description of the sample having access to the questionnaire | Open survey versus closed survey | Closed survey aimed at potential users of the dashboard |
|  | Contact mode | Email |
|  | Advertising the survey | Closed survey directed at specific respondents so no advertisement of the survey was done |
| Survey Administration | Web/E-mail | The survey was emailed to respondents via google forms and attached as a pdf for those that wished it in that format |
|  | context | A google drive folder was used for the project so google forms were a suitable method of collecting data |
|  | Mandatory/Voluntary | Surveys were Voluntary |
|  | Incentives | Respondents were possible end users of the developed application |
|  | Time/Date | The data was collected between March 2021 and June 2022 |
|  | Randomization of items or questionnaires | Questions were not randomised |
|  | Adaptive questioning | None of the questions were required. |
|  | Number of Items | The items in each survey ranged from 10 - 16 items. |
|  | Number of Pages | Each survey was between 3-5 pages |
|  | Completeness check | None. This was a voluntary survey with no required questions. |
|  | Review step | Respondents could edit survey until the final completion date. |
| Response Rates | Unique site visitor | N/A |
|  | View rate (Ratio of unique survey visitors/unique site visitors) | The survey is voluntary. The system cannot record the number of unique visitors, so the view rate  Respondents also queried their colleagues on surveys so an accurate number of viewers is not possible |
|  | Participation rate (Ratio of unique visitors who agreed to participate/unique first survey page visitors) | Rate cannot be cancelled as number of viewers cannot be accurately calculated |
|  | Completion rate (Ratio of users who finished the survey/users who agreed to participate) | This was a voluntary questionnaire. By default, the participants submit questionnaires on behalf of their consent to participate, so the completion rate cannot be calculated |
| Preventing multiple entries from the same individual | Cookies Used | None |
|  | IP check | None |
|  | Log File Analysis | N/A |
|  | Registration | N/A |
| Analysis | Handling of incomplete questionnaires | All surveys were analysed. No questions were mandatory. All feedback was considered |
|  | Questionnaires submitted with an atypical timestamp | Response data had a timeline. Surveys submitted after that time were considered where appropriate at a later date. |
|  | Statistical Correlation | N/A |
