# Supplementary material for: Building and Developing a Tool (PANDEM-2 Dashboard) to Strengthen Pandemic Management: Participatory Design Study
Source: JMIR Public Health Surveill. 2025 Mar 5;11:e52119. doi: 10.2196/52119 (PMC11923449; doi:10.2196/52119)
Supplement: Multimedia Appendix 7 [file publichealth_v11i1e52119_app7.docx]

| **Epic / User Story** | | |
| --- | --- | --- |
| As a policy advisor/health care professional I want to be able to see the number of cases, stratified and broken down by age, sex, time, area, and severity. I want to be able to monitor the situation, see clusters, and get an overview of how the virus is spreading over time. | | |
| **Overview** | | |
|  | A detailed breakdown of the number of cases over time and in which areas is important. Cases should be filterable by a variety of characteristics where possible; including age, sex, underlying conditions, subgroup, and the type of the pathogen | |
| **Use Case** | **Data Families** | **Geographical Elements** |
| Case Numbers | Cases | NUTS3 |
|  | Patients | National |
|  |  | Municipal |
|  |  | Individual |

| **Query** | | | |
| --- | --- | --- | --- |
| Number of Cases | | | |
| **Functional Category** | | | |
|  | Situational Awareness | | |
|  | Scenarios | | |
| **Query Filters** | **Time** | **Area** | **Query Resources** |
|  | Daily | Local | Total Number |
|  | Weekly | Regional | With proven infection |
|  | Monthly | National | With suspected infection |
|  | Yearly | NUTS | Severity of cases |
|  |  |  | Genetic subtype of pathogen |
|  |  |  | New cases over time/Incidence rates |
|  |  |  | With isolation status |
|  |  |  | 7 day incidence per 100,000 |
|  |  |  | Cluster by T-SNE analysis |
|  |  |  | Characteristics of patients (monitor outbreak and determine at risk groups) |
|  |  |  | Stratified by age |
|  |  |  | Stratified by sex |
|  |  |  | Underlying Condition |
|  |  |  | Stratified over time |
|  |  |  | Change in trend |
|  |  |  | Vulnerable groups |
|  |  |  | Effect of treatment |
|  |  |  | Personal Information |

Primary Query on left, followed by category, links to original user requirements document, and query resources.

*T-SNE is a statistical method for visualising high dimensional data

*The NUTS classification (Nomenclature of territorial units for statistics) is a hierarchical system for dividing up the economic territory of the EU.
