# Supplementary material for: Building and Developing a Tool (PANDEM-2 Dashboard) to Strengthen Pandemic Management: Participatory Design Study
Source: JMIR Public Health Surveill. 2025 Mar 5;11:e52119. doi: 10.2196/52119 (PMC11923449; doi:10.2196/52119)
Supplement: Multimedia Appendix 8 [file publichealth_v11i1e52119_app8.docx]

| **Appendix 8: Data availability and prioritisation survey questions** | |
| --- | --- |
| **General Data Topic** | **Question** |
| Available in your institution | Please indicate the most detailed available level for each variable/item in your institution. This would be the data you could upload on a local Pandem installation. |
| Publicly available level of detail | Please indicate the most detailed publicly available level. This would be the data you could upload on a shared Pandem installation. |
| Periodicity | Please indicate the most detailed reporting periodicity available for each variable/item. |
| TESSY/ECDC Format | Please indicate whether each variable/item is available at your institution in TESSY/ECDC format |
| Pathogens | Please indicate the pathogens for which this variable is collected in your institution. If other than COVID-19 and Influenza, please add comments |
| Dashboard priority | Please indicate the priority level you see in using this variable/item on PANDEM-2 dashboards |
| Forecasting priority | Please indicate the priority level you see in using this variable/item for PANDEM-2 prediction capabilities. |
| Resource planning priority | Please indicate the priority level you assign to using this variable/item for resource planning in PANDEM-2 |
