# Supplementary material for: Building and Developing a Tool (PANDEM-2 Dashboard) to Strengthen Pandemic Management: Participatory Design Study
Source: JMIR Public Health Surveill. 2025 Mar 5;11:e52119. doi: 10.2196/52119 (PMC11923449; doi:10.2196/52119)
Supplement: Multimedia Appendix 9 [file publichealth_v11i1e52119_app9.docx]

| **Variable Category** | **List of variable requirements** | **Variable Type** |
| --- | --- | --- |
| 01. Cases | Confirmed cases | Observation |
| 01. Cases | Suspected cases (possible, probable and unclassified) | Observation |
| 01. Cases | Cases per variant | Characteristic |
| 01. Cases | Cases per (age, sex, comorbidities) | Characteristic |
| 01. Cases | Incidence rates (last week, two weeks, month, and other not known potential time period) | Indicator |
| 01. Cases | Incidence rates (age, sex, comorbidities, variant) | Indicator |
| 01. Cases | Rt number | Indicator |
| 01. Cases | PPE Protective equipment (stock, type & need) | Resources |
| 01. Cases | Outbreak Id (if associated to known outbreak) | Observation |
| 01. Cases | N° of patients per severity level | Characteristic |
| 01. Cases | N° and proportion of imported cases | Observation |
| 02. Deaths | N° of deaths by X | Observation |
| 02. Deaths | N° of deaths | Observation |
| 02. Deaths | Mortality rates (age, sex, comorbidities) | Indicator |
| 02. Deaths | Mortality rates (with X) (last week, two weeks, month and other not known potential time period) | Indicator |
| 02. Deaths | Mortality rates with X (age, sex, comorbidities, variant) | Indicator |
| 03. Patients | N° of infected patients per bed type (clinic care, ICU, ventilator) | Observation |
| 03. Patient | N° of non-infected patients per bed type | Observation |
| 03. Patient | Length of stay | Observation |
| 03. Patient | Patient current status (recovered, death, still in care) | Observation |
| 03. Patient | N° of patients per facility type (hospital / LTHF / primary care and other not known potential values) | Characteristic |
| 03. Patient | Treatment received | Characteristic |
| 03. Patient | Patients potential risk factors (age, gender, comorbidities, municipality of residence) | Characteristic |
| 03. Patient | Hospital staff | Resources |
| 03. Patient | Patient resources: Beds, ventilators, oxygen, medicines, disinfection materials, ICU supplies... | Resources |
| 03. Patient | Hospital staff type e.g., intensive care, emergency care, etc... | Resources |
| 03. Patient | Bed type (ICU, regular clinical wards, emergency) | Resources |
| 03. Patient | Primary care staff | Resources |
| 03. Patient | Beds/Room occupancy and types (e.g., isolation) | Resources |
| 03. Patient | Vaccination Status | Observation |
| 04.Tests | Number of tests performed by type | Observation |
| 04.Tests | Link between test results and epidemiological surveys | Observation |
| 04.Tests | Test type details (brand and characteristics) | Characteristic |
| 04.Tests | Test results (positive, negative, unknown, pending) | Characteristic |
| 04.Tests | Positivity rate | Indicator |
| 04.Tests | Test resources (staff, supplies) | Resources |
| 05. Vaccination | Doses injected | Observation |
| 05. Vaccination | people that have received at least one dose | Observation |
| 05. Vaccination | People fully vaccinated | Observation |
| 05. Vaccination | Doses scheduled and target population | Observation |
| 05. Vaccination | Doses injected by age group, risk group, and brand/type | Characteristic |
| 05. Vaccination | Doses by vendor, batch | Characteristic |
| 05. Vaccination | Doses injected by occupation (HCW and other essential professionals and other not known potential values...) | Characteristic |
| 05. Vaccination | Doses injected in high-risk individuals - potential risk factors (immunosuppressed, comorbidities, pregnant women, elderly and other not known potential factors) | Characteristic |
| 05. Vaccination | Vaccination Side effects AEFI observed and severity | Characteristic |
| 05. Vaccination | Vaccination progress (proportion of vaccinated, overall, by age and risk group) | Indicator |
| 05. Vaccination | Vaccination resources (Staff, centres, supplies) | Resources |
| 06. Contact tracing | N° of index cases studied | Observation |
| 06. Contact tracing | Nº of contacts, secondary and tertiary cases per index case | Indicator |
| 06. Contact tracing | N° of clusters found (and cluster type - definition) | Characteristic |
| 06. Contact tracing | Confirmed cases that had travel during infectious period | Observation |
| 06. Contact tracing | Types of contact | Characteristic |
| 06. Contact tracing | Contact tracing details at individual level: travel, contacts, date of isolation, date of quarantine, transmission chains and other not known potential variables... | Observation |
| 06. Contact tracing | Cluster identification and characterisation | Characteristic |
| 06. Contact tracing | Notification delay (onset of symptoms - notification date) | Indicator |
| 06. Contact tracing | Contact tracers (staff working in contact tracing) | Resources |
| 07. Lab | Number of tests performed (overall and by individual) | Observation |
| 07. Lab | Speed of spread of variants (proportion among overall cases) | Observation |
| 07. Lab | seroprevalence (and test type) | Indicator |
| 07. Lab | Seaway water virus presence (and levels) | Observation |
| 07. Lab | Mutations/Sequences spread and distributions | Characteristic |
| 07. Lab | link lab data with cases/patient data | Characteristic |
| 07. Lab | link with aggregated epidemiological data | Characteristic |
| 07. Lab | Sensibility & specificity of test methods | Document |
| 08. Emergency calls | Monitoring number of emergency calls (overall and by syndrome) | Observation |
| 08. Emergency calls | Comparison current situation with peacetime symptoms, notifications and diagnostic rates. | Observation |
| 08. Emergency calls | Severity of victims (at call and scene) | Characteristic |
| 08. Emergency calls | N° of calls from people declared as confirm case | Observation |
| 08. Emergency calls | Monitoring of symptoms from emergency calls | Characteristic |
| 09. First response | Ongoing emergencies (types) | Observation |
| 09. First response | Visits to general practitioner (GP) with compatible symptoms (disease X) | Observation |
| 09. First response | Details/type of protocol applied | Characteristic |
| 09. First response | Public health Staff (surveillance, prevention and control activities and other not known potential activities...) | Resources |
| 09. First response | Emergency Staff | Resources |
| 10. Transport | Patient transportation type (for suspicious or confirmed cases) | Observation |
| 10. Transport | Current ambulance activity | Observation |
| 10. Transport | Number of patients transferred | Observation |
| 10. Transport | Transport statistics (duration, times) | Characteristic |
| 10. Transport | Transport resources (ambulances) | Resources |
| 10. Transport | Ambulances / type | Resources |
| 10. Transport | patient transport threshold | Resources |
| 10. Transport | patient transport protocols | Document |
| 11. Measures | N° of people entering to the country (by country of origin, and city-airport of entrance) | Observation |
| 11. Measures | Mitigation measures and policies | Observation |
| 11. Measures | Prevention or control measure details:   - type (e.g., lockdown) - start - end - place (place or geographical location) | Characteristic |
| 11. Measures | Border rules/laws | Document |
| 12. Population study | Adherence to prevention and control measures | Observation |
| 12. Population study | Is people understanding public health communication | Observation |
| 12. Population study | Alerts & Early warning signals | Indicator |
| 12. Population study | Social media custom analysis | Indicator |
| 12. Population study | Vaccination acceptance willingness | Indicator |
| 12. Population study | level of trust in the Government and institutions | Indicator |
| 12. Population study | Measure social impact (psychological, lifestyle) | Indicator |
| 12. Population study | indirect impact on health (other notifiable disease, disruption of services, indirect deaths and morbidity...) | Indicator |
| 12. Population study | People beliefs and opinions on pandemic | Indicator |
| 12. Population study | Most consulted public information sites | Observation |
| 12. Population study | People information needs | Document |
| 13. Referentials | Denominators for potential risk factors or individuals at risk | Referential |
| 13. Referentials | pathogen specific referential epidemiological parameters  (Host, vector, latency, contagiousness, Serial interval, Susceptibility...) | Referential |
| 13. Referentials | Symptoms & signs by pathogen | Referential |
| 13. Referentials | Care procedures (for new diseases) | Referential |
| 13. Referentials | Variants (VOI, VOC): variant of interest (VOI), variant of concern (VOC) according to WHO and ECDC indications | Referential |
| 13. Referentials | denominators and maps for different Geographic location (local to international) | Referential |
| 13. Referentials | Population denominators (age, sex, country of birth, vaccination status, comorbidities, age group ...) | Referential |
| 13. Referentials | Social determinants by area or case (country of birth, wealth, studies, occupation...) | Referential |
| 13. Referentials | Care providers by area | Referential |
| 13. Referentials | User shared guidelines | Document |
| 13. Referentials | Places of infection | Document |
| 13. Referentials | supplies for potential or confirmed effective medication | Document |
| 14. Metadata | Variable definitions (calculation method, description) | Referential |
| 14. Metadata | Source contact | Referential |
| 14. Metadata | Data owner | Referential |
| 14. Metadata | Data providers for dashboards | Referential |
| 14. Metadata | Dashboard profile e.g., emergency | Referential |
| 14. Metadata | GDPR compliance | Referential |
