# Supplementary figures and images for: Building and Developing a Tool (PANDEM-2 Dashboard) to Strengthen Pandemic Management: Participatory Design Study
Source: JMIR Public Health Surveill. 2025 Mar 5;11:e52119. doi: 10.2196/52119 (PMC11923449; doi:10.2196/52119)

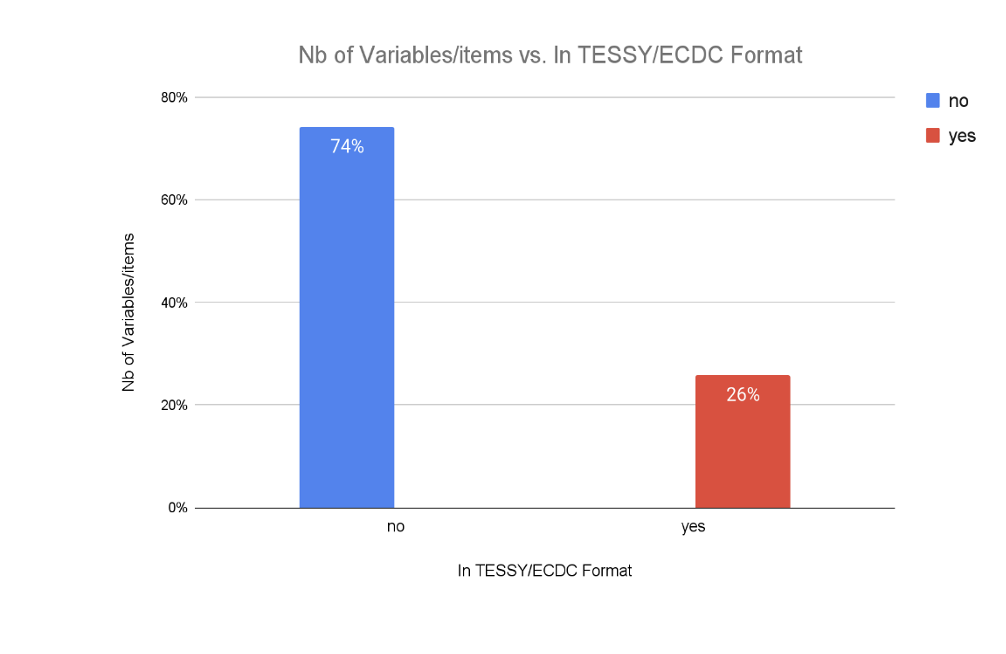

Supplement: Multimedia Appendix 10 [file publichealth_v11i1e52119_app10.png]
