# Supplementary material for: Building and Developing a Tool (PANDEM-2 Dashboard) to Strengthen Pandemic Management: Participatory Design Study
Source: JMIR Public Health Surveill. 2025 Mar 5;11:e52119. doi: 10.2196/52119 (PMC11923449; doi:10.2196/52119)
Supplement: Multimedia Appendix 11 [file publichealth_v11i1e52119_app11.pdf]

PANDEM-2
PANDEMIC PREPAREDNESS AND RESPONSE

Surveillance | Healthcare Capacity | Contact Tracing | Interventions | Disease Profile | Vaccines | Scenarios

1

HOSPITALISATIONS

A rise in the number of patients with COVID-19 could overburden hospitals. This number is also a valid indicator for epidemic growth. This section of the dashboard shows the number of patients with COVID-19 admitted to hospital and the number of regular hospital beds occupied by patients with COVID-19.

Last values obtained on Tuesday, 9 November. Is updated on a daily basis.

Source: NICE via RIVM & LCPS

2

Hospitalisations

Bed Occupancy

Source: Organisation – Last Update: 04.11.2021, 07.52h

The published data is based on reports that were sent to the CMS by hospitals and clinics with intensive care units via the Information and Operations System (IOS). The data is processed in such a way that reports are valid for a maximum of 7 days, unless updates are received.
Due to the different reporting times and procedures, the figures shown here may differ from those published by other institutions. Due to mathematical rounding, the sum of the percentages may not be exactly 100%.

Number of operable beds	1,34000
non-ICU	1,34000
ICU	1,34000
Emergency	1,34000
Bed occupancy	1500 (75%)
Bed occupancy with pathogen-x	750 (50%)
Last 7 days	789
	▲ 131 (13%)
Median length of stay	1.7

Hospitalisations

Bed Occupancy

Source: Organisation – Last Update: 04.11.2021, 07.52h

Number of operable beds	1,34000
non-ICU	1,34000
ICU	1,34000
Emergency	1,34000
Bed occupancy	1500 (75%)
Bed occupancy with pathogen-x	750 (50%)
Last 7 days	789
	▲ 131 (13%)
Median length of stay	1.7

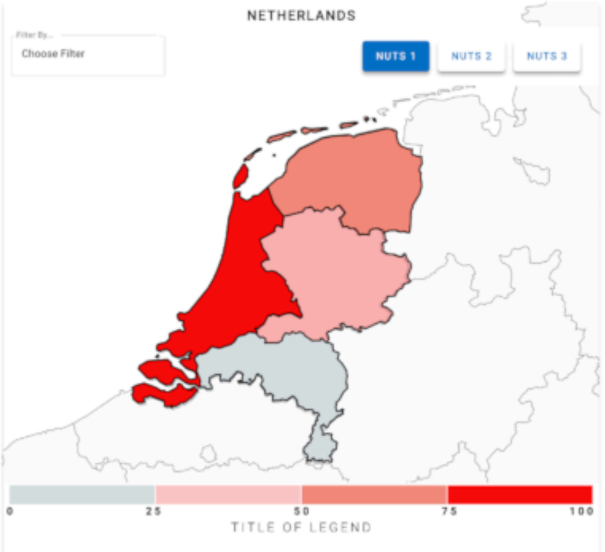

3

4

Hospitalisations: Admissions & Bed Occupancy

ADMISSIONS ICU

A rise in the number of patients with COVID-19 could overburden hospitals. This number is also a valid indicator for epidemic growth. This section of the dashboard shows the number of patients with COVID-19 admitted to hospital and the number of regular hospital beds occupied by patients with COVID-19.

Last values obtained on Wednesday, 10 November. Is updated on a daily basis.
Source: NICE via RIVM & LCPS

ALL | 6 MONTHS | 3 MONTHS | 4 WEEKS | 2 WEEKS | LINEAR | LOG

DAILY | WEEKLY | CUMULATIVE | ABSOLUTE NUMBERS | PER 100,000 | PROPORTION (%)

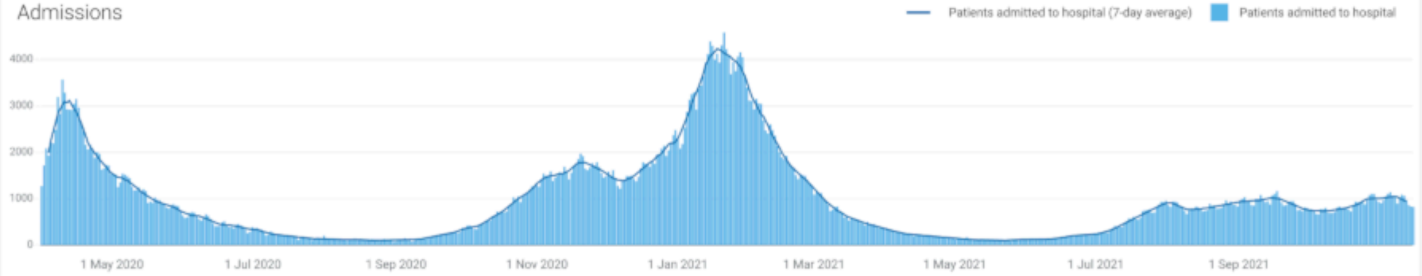

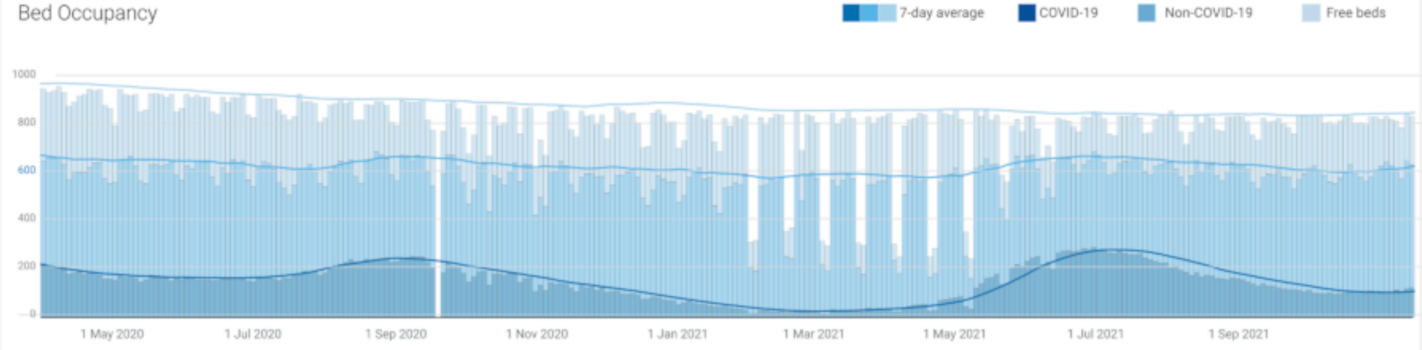

DOWNLOAD DATA | DOWNLOAD IMAGE | VIEW AS TABLE

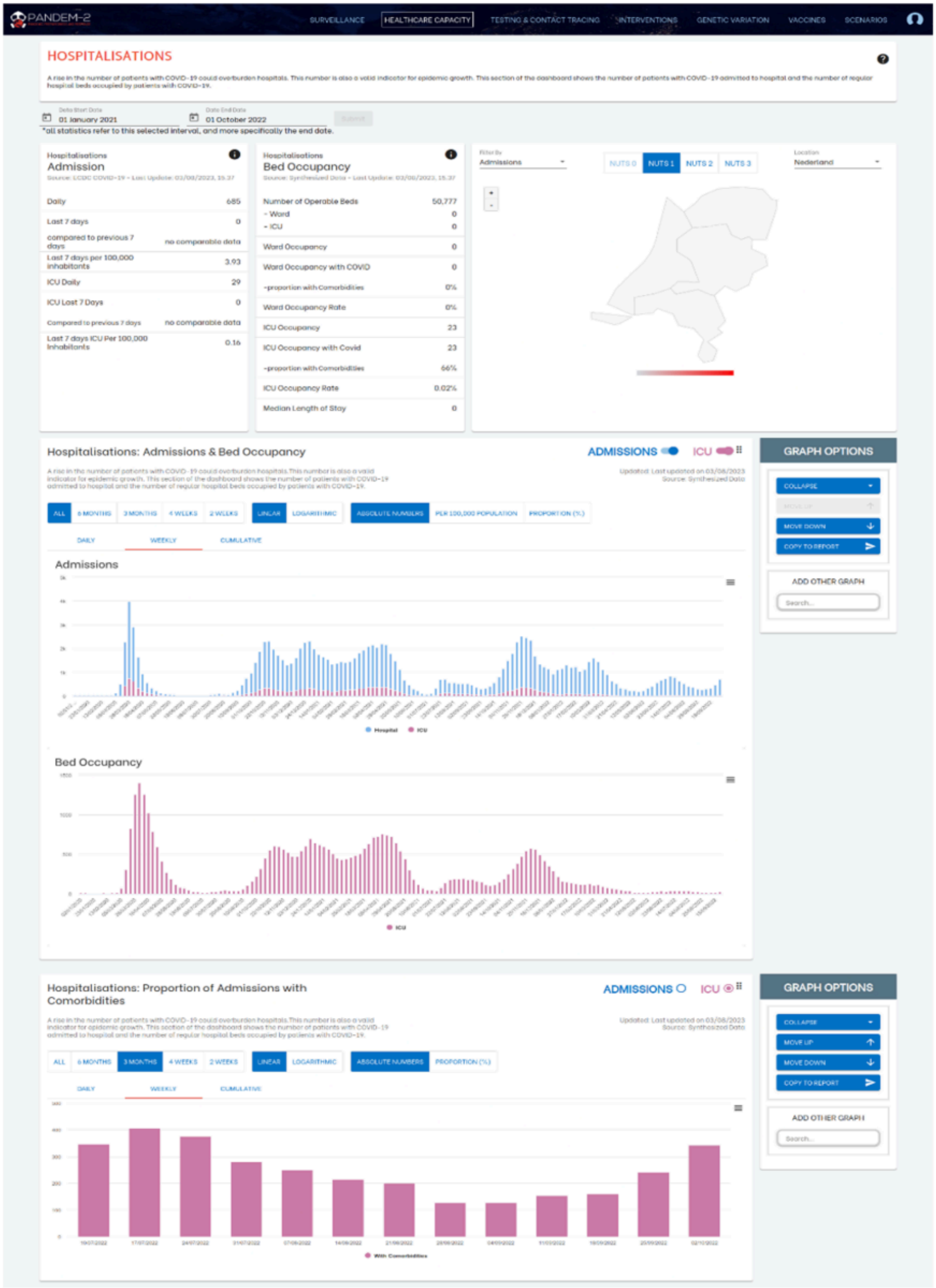
PANDEM-2
SURVEILLANCE
HEALTHCARE CAPACITY
TESTING & CONTACT TRACING
INTERVENTIONS
GENETIC VARIATION
VACCINES
SCENARIOS
HOSPITALISATIONS
A rise in the number of patients with COVID-19 could overburden hospitals. This number is also a valid indicator for epidemic growth. This section of the dashboard shows the number of patients with COVID-19 admitted to hospital and the number of regular hospital beds occupied by patients with COVID-19.
01 January 2021
01 October 2022
*all statistics refer to this selected interval, and more specifically the end date.
Hospitalisations
Admission
Daily 685
Last 7 days 0
compared to previous 7 days no comparable data
Last 7 days per 100,000 inhabitants 3.93
ICU Daily 29
ICU Last 7 Days 0
Compared to previous 7 days no comparable data
Last 7 days ICU Per 100,000 inhabitants 0.16
Hospitalisations
Bed Occupancy
Number of Operable Beds 50,777
- Ward 0
- ICU 0
Ward Occupancy 0
Ward Occupancy with COVID 0
-proportion with Comorbidities 0%
Ward Occupancy Rate 0%
ICU Occupancy 23
ICU Occupancy with Covid 23
-proportion with Comorbidities 66%
ICU Occupancy Rate 0.02%
Median Length of Stay 0
Admissions
NUTS 0
NUTS 1
NUTS 2
NUTS 3
Nederland
Hospitalisations: Admissions & Bed Occupancy
ADMISSIONS
ICU
GRAPH OPTIONS
COLLAPSE
MOVE UP
MOVE DOWN
COPY TO REPORT
ADD OTHER GRAPH
ALL
6 MONTHS
3 MONTHS
4 WEEKS
2 WEEKS
LINEAR
LOGARITHMIC
ABSOLUTE NUMBERS
PER 100,000 POPULATION
PROPORTION (%)
DAILY
WEEKLY
CUMULATIVE
Admissions
Hospital
ICU
Bed Occupancy
ICU
Hospitalisations: Proportion of Admissions with Comorbidities
ADMISSIONS
ICU
GRAPH OPTIONS
COLLAPSE
MOVE UP
MOVE DOWN
COPY TO REPORT
ADD OTHER GRAPH
ALL
6 MONTHS
3 MONTHS
4 WEEKS
2 WEEKS
LINEAR
LOGARITHMIC
ABSOLUTE NUMBERS
PROPORTION (%)
DAILY
WEEKLY
CUMULATIVE
With Comorbidities
