# Supplementary material for: Building and Developing a Tool (PANDEM-2 Dashboard) to Strengthen Pandemic Management: Participatory Design Study
Source: JMIR Public Health Surveill. 2025 Mar 5;11:e52119. doi: 10.2196/52119 (PMC11923449; doi:10.2196/52119)
Supplement: Multimedia Appendix 12 [file publichealth_v11i1e52119_app12.docx]

**Appendix 11: Illustrations of initial and final dashboard designs: Progression of the map component**

**
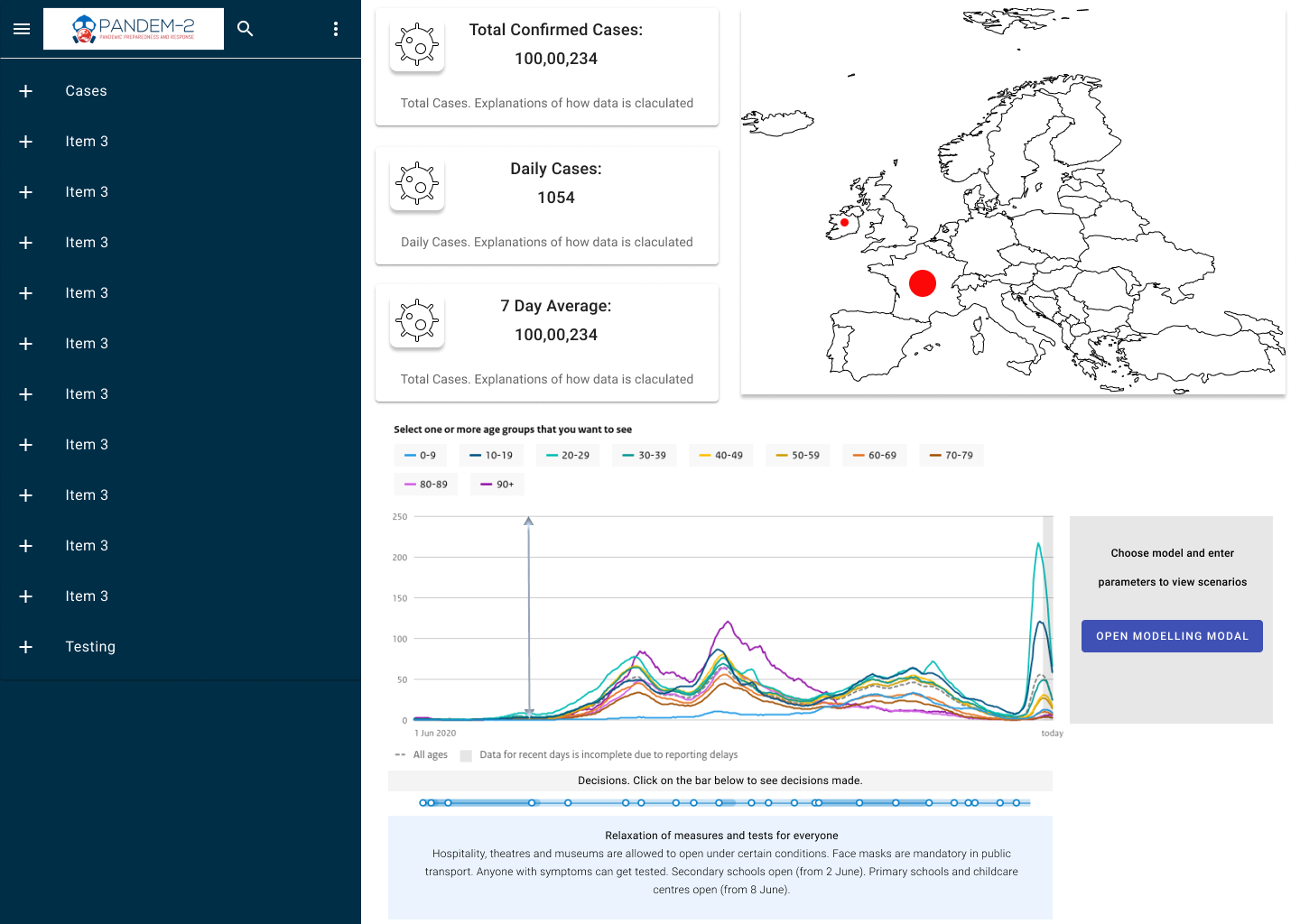
**

***Initial draft on how a dashboard page might be structured - Page contains menu, a number of indicators and a map of Europe. Below this is a graph that could highlight further information related to an indicator***

**
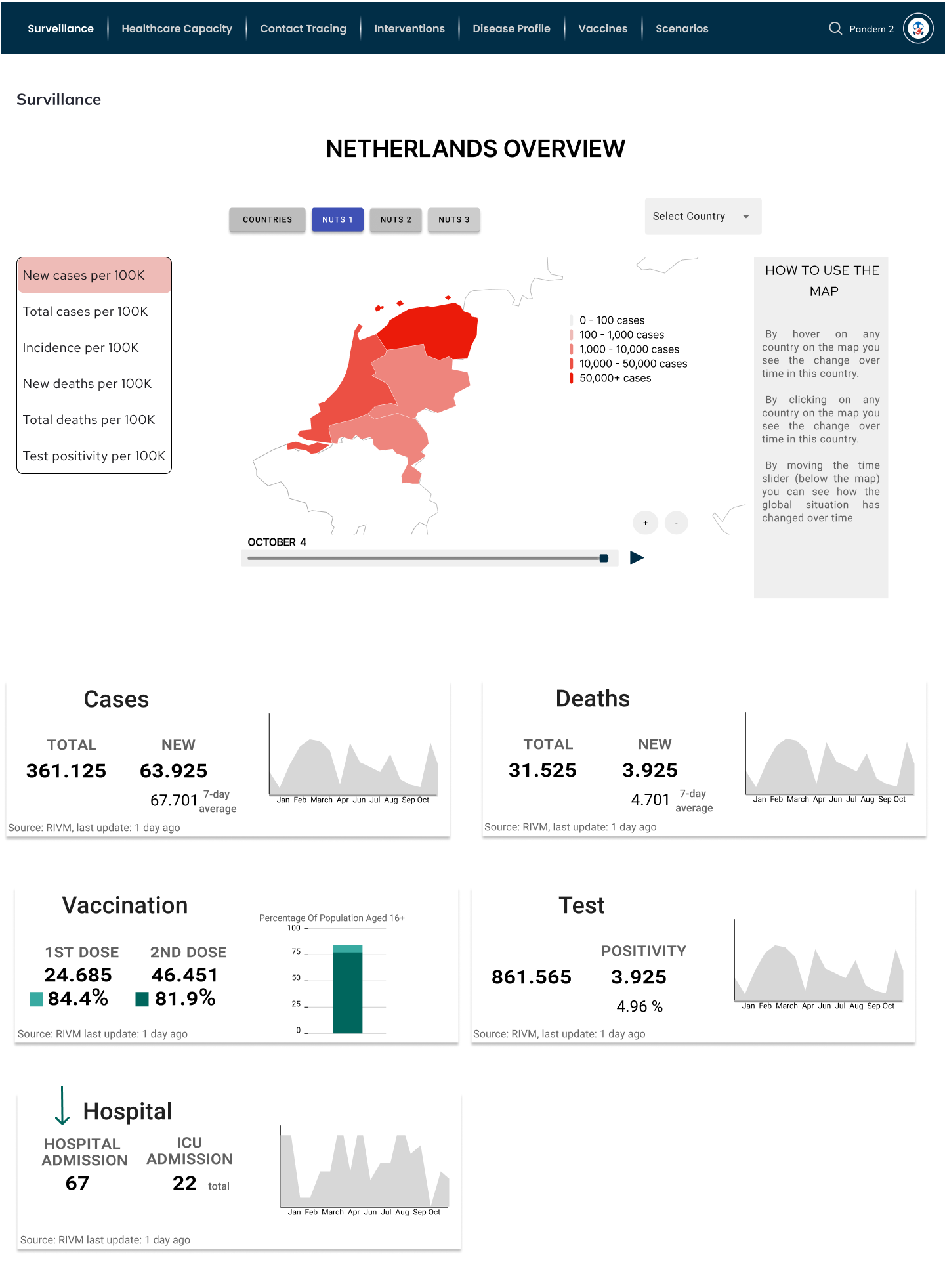
**

***Another draft on how a dashboard page might be structured, this one for a specific region. The map has been centered and can show different variables. The map has a timeline below it. Indicators in this example are below the map.***

**
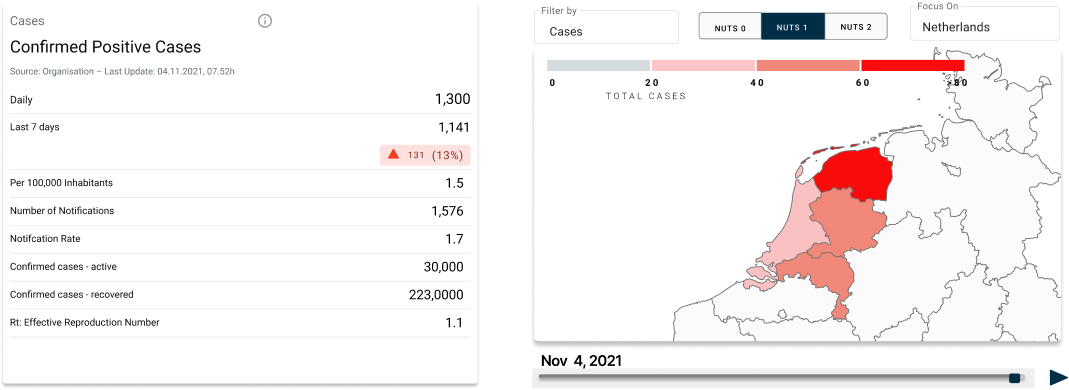
**

***Idea of indicator card component alongside map component. Expanding the idea of indicators into an indicator card with high level data based on that indicator. Map has data, NUTS, and region filters as well as the timeline.***

**
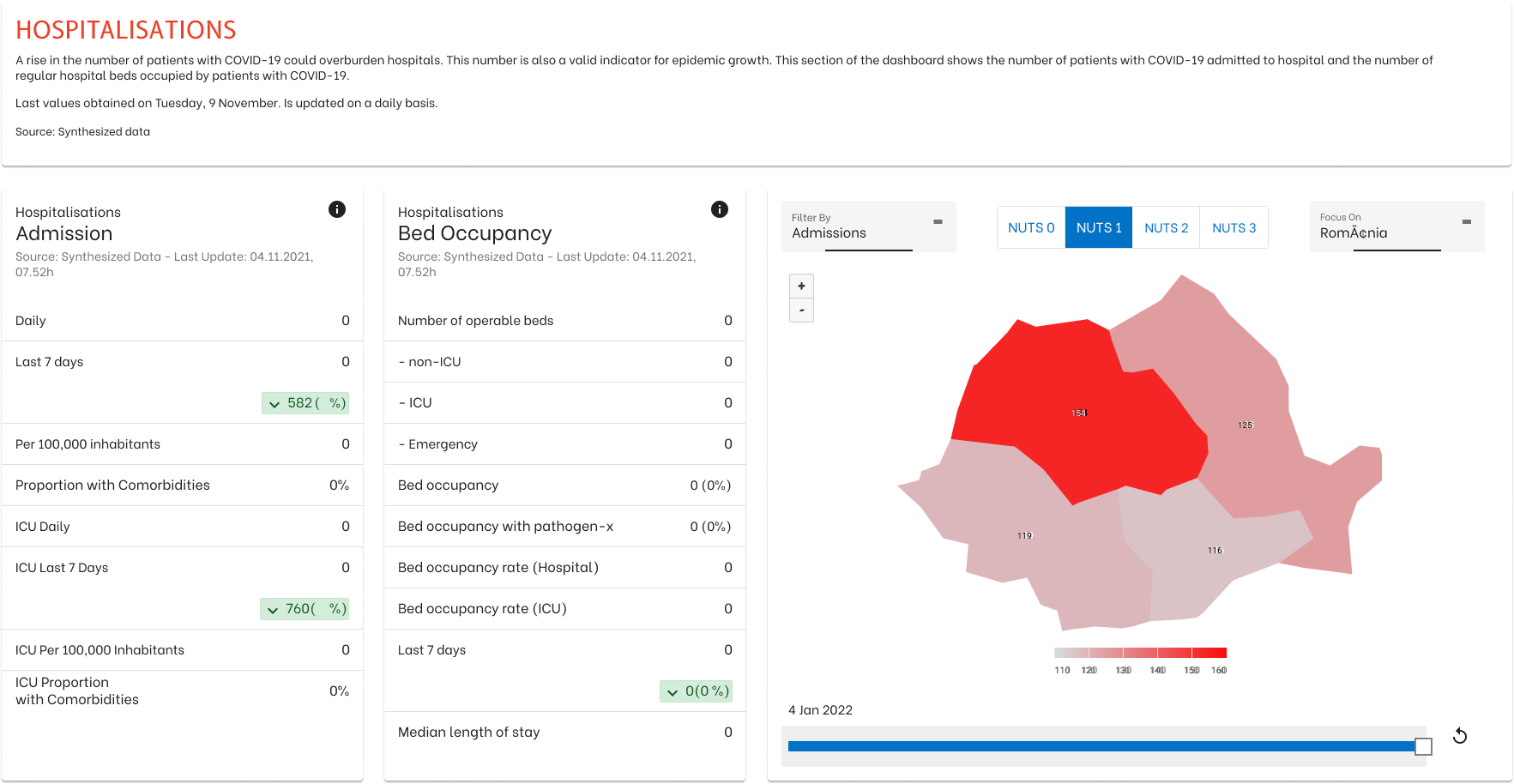
**

***Final designs of how indicator cards and map are presented side by side. Page can have one or two indicator card components depending on data being presented. Map has multiple filters, data, NUTS, and location. Map component also has timeline slider at the bottom of the component***
