# Supplementary material for: Building and Developing a Tool (PANDEM-2 Dashboard) to Strengthen Pandemic Management: Participatory Design Study
Source: JMIR Public Health Surveill. 2025 Mar 5;11:e52119. doi: 10.2196/52119 (PMC11923449; doi:10.2196/52119)
Supplement: Multimedia Appendix 13 [file publichealth_v11i1e52119_app13.docx]

# GUIDED Checklist

| **Question** | **Response** | **Page Numbers** |
| --- | --- | --- |
| 1. Report the context for which the intervention was developed. | The PANDEM-2 dashboard was developed to address challenges encountered during the COVID-19 pandemic, particularly in real-time data sharing, data standardisation, and decision-making. The tool aims to enhance cross-border collaboration and improve pandemic management. These issues were significant across Europe due to fragmented data and varied response strategies. | 1-3 |
| 1. Report the purpose of the intervention development process. | The purpose of the development was to create a decision-making tool that could improve pandemic preparedness and response management through effective data integration, visualisation, and collaboration across European countries. | 1-3 |
| 1. Report the target population for the intervention development process. | The target population includes public health officials, healthcare workers, and pandemic managers across EU member states. The dashboard is designed to support these users in their decision-making processes during pandemics. | 3, 8 |
| 1. Report how any published intervention development approach contributed to the development process. | The development process followed a participatory design approach, incorporating end-user feedback iteratively. This allowed users to engage in the design and refinement of the dashboard, ensuring the tool was user-centered and met the needs of public health professionals. | 8-12 |
| 1. Report how evidence from different sources informed the intervention development process. | The development process was informed by a review of 25 publicly available COVID-19 dashboards. This review helped identify gaps in current pandemic dashboards and informed the selection of key data variables and indicators. | 6-7 |
| 1. Report how/if published theory informed the intervention development process. | The intervention development was grounded in practical, data-driven needs rather than a formal theoretical model. The dashboard’s design was based on the real-world challenges faced by public health professionals during health emergencies. | 6, 8-11 |
| 1. Report any use of components from an existing intervention in the current intervention development process. | The development incorporated features from existing pandemic dashboards, such as data visualisation formats and report generation tools, which were adapted to meet the specific needs of PANDEM-2 users. Customisation options were included based on feedback from healthcare professionals. | 19-21 |
| 1. Report any guiding principles, people, or factors that were prioritized when making decisions during the intervention development process. | User-friendliness, data security, and scalability were key guiding principles. The involvement of end-users in workshops and feedback sessions played a significant role in shaping the dashboard’s design, especially regarding customisable data views and privacy protection. | 9-13 |
| 1. Report how stakeholders contributed to the intervention development process. | Stakeholders, including public health professionals from 13 EU countries, contributed through workshops and participatory design sessions. They provided feedback on key features, data variables, and the interface, ensuring the dashboard met their real-world needs. | 9-13 |
| 1. Report how the intervention changed in content and format from the start of the intervention development process. | Based on user feedback, the dashboard underwent several changes. For instance, customisable report generation and the ability to filter data across different time frames were added. Users also emphasised the importance of diverse data visualisation formats, which were incorporated into the final design. | 19-24 |
| 1. Report any changes to interventions required or likely to be required for subgroups. | While the dashboard was designed for public health professionals, it did not undergo significant subgroup adjustments. However, some features were adapted to address varying data standardisation needs across different countries, as identified during the development process. | 19-24 |
| 1. Report important uncertainties at the end of the intervention development process. | Several uncertainties remain at the end of the PANDEM-2 dashboard's development process, particularly around data standardisation across EU member states, which affects the consistency and reliability of shared data. Additionally, data-sharing policies and privacy concerns across borders could challenge the full integration and functionality of the dashboard. Finally, the dashboard’s scalability to future pandemics remains to be tested. | 27-28 |
| 1. Follow TIDieR guidance when describing the developed intervention. | The PANDEM-2 dashboard’s description provides detailed information on its development process, including the participatory approach, iterative design, and user feedback incorporation. The dashboard’s architecture, data collection methods, and security measures are described extensively. Additionally, the modular structure and customisable data visualisation features are outlined in depth. The system is documented with sufficient technical and methodological details to facilitate replication and adaptation for future pandemic preparedness projects. | 3-24 |
| 1. Report the intervention development process in an open-access format. | The paper documents the development process in a fully transparent manner and includes links to demo videos of the dashboard prototype and supplementary materials hosted on Zenodo, an open-access platform. Additionally, key data and methodological details are shared to promote replicability and wider adoption. | 3-14, 28, 32-52 |
